# Local perception of ecosystem services and their conservation in Sudanian savannas of Burkina Faso (West Africa)

**DOI:** 10.1186/s13002-022-00508-w

**Published:** 2022-02-19

**Authors:** Assétou Nabaloum, Dethardt Goetze, Amadé Ouédraogo, Stefan Porembski, Adjima Thiombiano

**Affiliations:** 1 Laboratory of Plant Biology and Ecology, University Joseph Ki-Zerbo, 03 BP 7021 Ouagadougou 03, Burkina Faso; 2 Institute of Biological Sciences, Department of Botany, University of Rostock, 18051 Rostock, Germany

**Keywords:** Indigenous knowledge, Land use, Plant vulnerability, Sustainable management, Sociocultural groups

## Abstract

**Context:**

In Burkina Faso, Sudanian savannas are important ecosystems for conservation of plant diversity. Due to desertification and insecurity, population migration from the North has increased human density and anthropogenic pressure on southern savannas. This study aims to investigate knowledge of local populations on ecosystem services (ES) and perception of their conservation.

**Method:**

Individual semi-structured interviews about knowledge on ES and ecosystem conservation issues were conducted. Informants were selected according to sociocultural groups and sex in three areas of different land use intensity: the communal area of Dano (CAD), the Total Wildlife Reserve of Bontioli (TWRB) and the Game Ranch of Nazinga (GRN). The use value and vulnerability index of each plant species were determined. A cluster analysis and a principal component analysis were carried out to identify the particular knowledge of different ethnic groups.

**Results:**

Overall, 163 plant species were cited for fifteen ES. Provisioning services were most frequently cited (100%), regulating services second most frequently (92.47%). Entire plants were exclusively used for ES with non-material benefits (protection against wind, for shading, soil fertility, erosion prevention, tourism and religion). The ten species contributing most to ES provision were *Vitellaria paradoxa*, *Parkia biglobosa*, *Diospyros mespiliformis*, *Adansonia digitata*, *Lannea microcarpa, Faidherbia albida*, *Khaya senegalensis*, *Afzelia africana, Ficus sycomorus*, *Pterocarpus erinaceus.* Seven of them were identified as highly vulnerable. Around GRN, migrants and natives shared the same knowledge, while migrants in TWRB used the ES only to a small extent due to restricted contact with the native population. Migrants and natives of GRN had more knowledge on tourism and crafts services while the natives of CAD and TWRB made use of the services that sustain the quality of the agricultural land and meet their primary needs. To reduce further degradation, different communities suggested unanimously raising awareness of the importance of biodiversity and ecosystem conservation. The most quoted motivations to preserve ecosystems were vegetation sustainability and village development.

**Conclusion:**

This study documented important local knowledge-based information to guide cultivation of local multipurpose species and initiation of communities to practice best management strategies for sustainable conservation of biodiversity.

## Background

Ecosystem services (ES) are defined as the goods and services obtained by the human population from ecosystems, directly or indirectly, to assure its well-being [1]. ES can be classified in four main categories. The supporting services derive from general functioning of an ecosystem, the regulating services correspond to the direct services of ecological functions on site, and provisioning and cultural services refer to direct services of obtaining goods and social and spiritual well-being from ecosystems. While the provisioning services provide finished products of ecosystems, the non-material cultural services allow for developing and enriching knowledge systems, social relationships and aesthetic values [2]. Products of ecosystems used for provisioning services (such as food, fodder, wood, medicinal compounds) include non-timber forest products (NTFP) such as fruits, leaves, seeds, flowers, bark, medicinal herbs, as well as wood cut from trees for supply of energy and for construction.

In West African semi-arid areas, local populations strongly depend on plant resources for meeting their daily needs [3–6]. Local people consider savanna ecosystems as their own good, as granary, pharmacy, pasture, place of religious worship and source of the strength of their territory [7]. Ecosystem functions and services do not only result from good ecosystem health, but also from the use that populations have made in various biogeographic and geo-economic contexts [8].

In Sudanian savanna ecosystems, climate and soil characteristics are favorable to the development of a diversified and dense vegetation cover [9]. In the semi-arid context of Burkina Faso, Sudanian savannas constitute a particular hot spot of plant species diversity [10, 11]. Here, Zizka et al. [10] recorded 71% of all plant species of the country, with more than half of them being rare. Recent migration of human populations from the North to the South, fleeing desertification and climate change consequences on arable lands, has caused population density to increase in Sudanian savannas. In addition, terrorist attacks in the northern and eastern regions of the country have caused a new wave of migration and a raise in population density from 27.3 to 51.66 inhabitants/km^2^ in the Southwest region between 1985 and 2019 [12]. This population growth has immediate consequences such as intensification of anthropogenic pressure on plant resources and protected areas [13]. Dimobe et al. [14 and 15] noted a significant decrease in natural vegetation from 1984 to 2013, followed by an expansion of croplands and habitat fragmentation in protected areas in the South Sudanian zone of the country [15].

Inclusive sustainable management of plant resources by the forest authority in collaboration with local populations could be a response to the strong anthropogenic pressure that ecosystems are facing, as the fundamental ecological role of forests is as important as their economic and social roles to local populations [3]. Indigenous people play a crucial role in biodiversity conservation through their traditional knowledge on species and habitats and their socioeconomic and symbolic practices as well [16]. However, many local populations suffer from consequences of large development projects and exploitation of natural resources. Among these consequences are land expropriation, loss of identity, language and culture [16]. Thus, local populations adapt their use to the change they perceive [8] in different ecosystems. They know that their survival essentially depends on their adaptation to the socio-environmental impact of climate change [17]. Better conservation of ecosystems requires good mastering of endogenous and scientific knowledge [7]. In addition, together with processes of decentralization and population self-management, the local scale is the relevant geographic and socio-economic space for conducting participatory development policies [18]. According to Holou and Sinsin [19], it would allow for answering one of the major concerns of African countries which is the rational and sustainable management of natural resources.

In the last decade, research questions on ES have been increasingly focused on provisioning services [4, 5, 20–25]. In order to design effective vegetation management that prevents damage and promotes well-being of local people, it is important to take into account the needs of the latter. Thus, the present study aims to (i) understand knowledge of local populations on ecosystem services provided by plant species, (ii) identify factors that influence this knowledge, and (iii) understand local perceptions of sustainable management of plant communities.

## Methods

### Study area

**Fig. 1:**
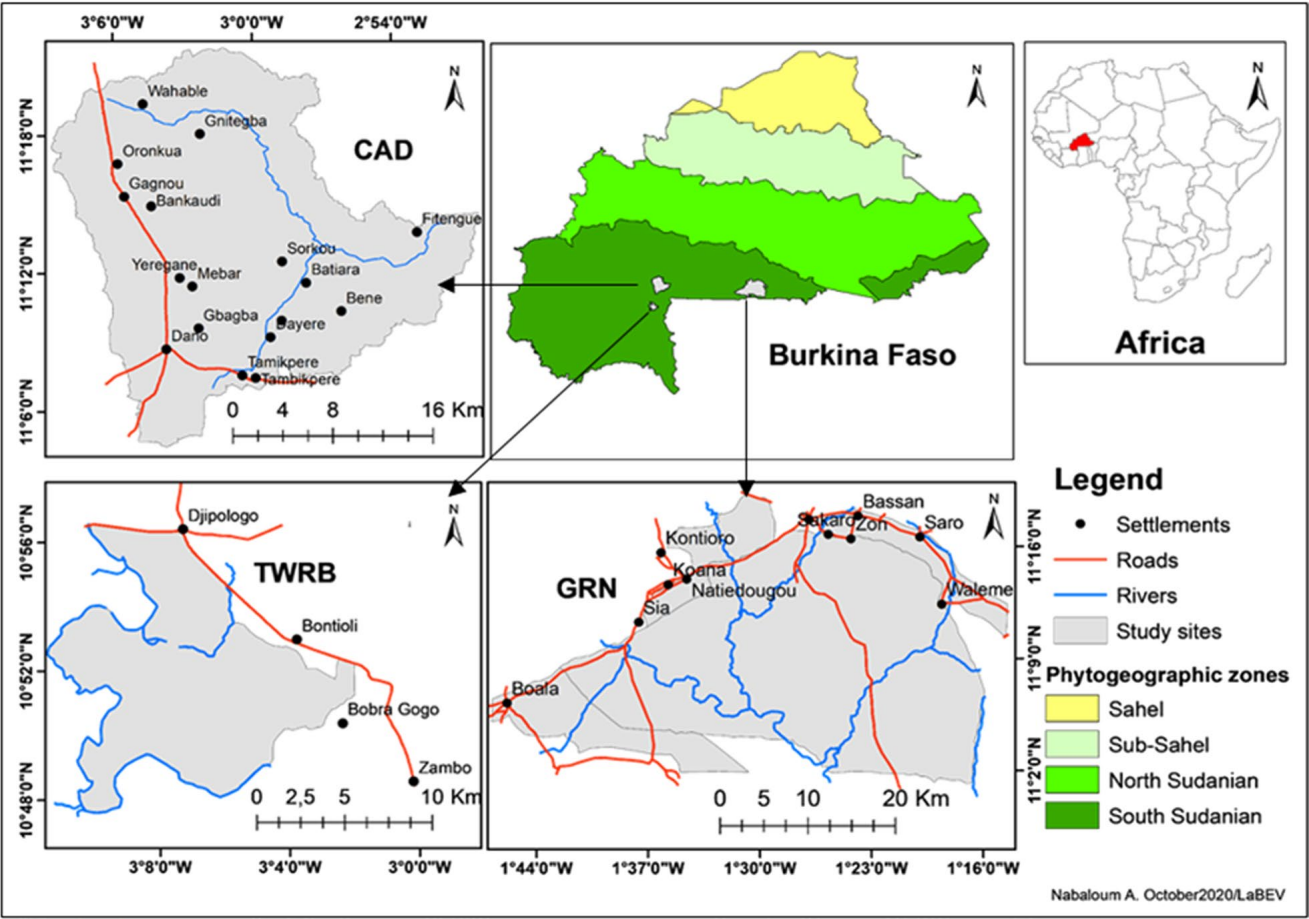
Location of the study sites in Burkina Faso. TWRB: Total Wildlife Reserve of Bontioli (medium use intensity); CAD: communal area of Dano (high use intensity); GRN: Game Ranch of Nazinga (low use intensity)

The study was carried out in the South Sudanian phytogeographical sector of Burkina Faso [9]. The sampling areas were chosen according to variations in land-use intensity and identified through classification of land-use/land cover data from multi-temporal Landsat images (for methodological details see [14, 15]), allowing for assigning them to a three-point scale with high, medium and low land-use intensity [26]. The communal area of Dano (CAD), located in the southwestern region of Burkina Faso and characterized by agrosystems and grazing land, corresponds to the high land-use intensity level (Fig. 1). The Total Wildlife Reserve of Bontioli (TWRB), also located in the southwestern region, consists of a protected area of IUCN category I under several human pressures, and corresponds to the medium land-use intensity level. The Game Ranch of Nazinga (GRN) and its ZOVICs (village hunting zones), which are protected areas of IUCN category VI, devoted to hunting and located in the South-central region, corresponds to the low land-use intensity level.

The vegetation types in the study area are grass savannas, shrub savannas, tree savannas, savanna woodlands, woodland and gallery forests [27]. Dominant woody species are *Vitellaria paradoxa* C.F. Gaertn, *Terminalia laxiflora* Engl. & Diels*, Terminalia macroptera* Guill. & Perr., *Combretum glutinosum* Perr. ex DC.*, Combretum micranthum* G. Don*, Combretum adenogonium* Steud. ex A.Rich., *Combretum collinum* Fresen., *Anogeissus leiocarpa* (DC.) Guill. & Perr., *Detarium microcarpum* Guill. & Perr., *Piliostigma thonningii* (Schum.) Milne-Redhead, *Isoberlinia doka* Craib & Stapf and *Lannea acida* A. Rich. The dominant grass species are *Andropogon gayanus* Kunth*, Hypparhenia rufa* (Nees) Stapf*, Loudetia togoensis* (Pilger) C.E. Hubbard and *Schizachyrium sanguineum* (Retz.) Alston [14, 15].

The climate of the study area is Sudanian with a unimodal rainy season of 5 to 6 months from May to September or October. The mean annual rainfall during a 30-year period (1986–2015) is 1048.73 ± 146.7 mm. The mean temperature for this same period is 28.1 ± 2.15 °C (National Direction of Meteorology of Burkina Faso).

The native sociocultural groups are the Dagara and the Pougouli in CAD, the Dagara in TWRB and the Kassena in GRN. The dominant non-native sociocultural group encountered in all sites are the Mossi which have moved to these areas to practice agriculture on more fertile soils and recently for gold panning. Agriculture, livestock and gold panning constitute the main human activities in the study area.

### Sampling design and data collection

#### Ethnobotanical data

Seventeen villages were randomly selected in the three study sites following a random sampling scheme: seven villages in CAD, five around TWRB and five around GRN. The selection of informants was stratified, based on sociocultural groups (Dagara, Pougouli, Mossi, Kassena) and sex [28]. The Mossi were immigrants and the other ones indigenous (Table 1). Individual semi-structured interviews were conducted from December 2016 to March 2017 to collect the data following a questionnaire. In each village and for each sociocultural group, ten informants (five men and five women were selected randomly) of at least 20 years of age were interviewed with their consent, yielding a total sample of 240 informants (Table 1). Interviews were conducted in the local language of the informants and translated by a local translator. Informants were asked to list the plant species they use and the ES rendered, and the used organs were recorded. Informants were also questioned about availability and dynamics of the plant species, reasons for these dynamics, solutions in case of regression and motivations to conserve biodiversity. Each informant classed the items of suggested solution and motivation by preference order. For identifying the plant species cited in local language, in each study site, the “walk-in-the woods” method was used at the end of the interviews. This method consisted in field visits with members of the community who have good knowledge about plant species [29]. They were selected for having cited the largest number of utilized species during the interview phase. During the field visit, fresh samples of cited species were collected and pressed for identification using the floras of Berhaut [30], Lebourgeois and Merlier [31], Poilecot [32] and the field handbook of Arbonnier [33]. The identified samples were verified by comparison with samples of the Ouagadougou herbarium at Joseph Ki-Zerbo University.

**Table 1:** Sociodemographic characteristics of the informants

Study sites	CAD	TWRB	GRN
Number of villages	7	5	5
Sociocultural group	Dagara (71%), Pougouli (29%)	Dagara (71%), Mossi (29%)	Kassena (50%), Mossi (50%)
Gender	Male (50%); Female (50%)	Male (50%); Female (50%)	Male (50%); Female (50%)
Age classes	Young (34%); Adult (42%); Elder (24%)	Young (31%); Adult (46%); Elder (23%)	Young (42%); Adult (38%); Elder (20%)

TWRB, Total Wildlife Reserve of Bontioli (medium use intensity); CAD, communal area of Dano (high use intensity); GRN, Game Ranch of Nazinga (low use intensity); Age classes, Young = [20–40[; Adult = [40–60[; Elder ≥ 60

#### Floristic data

A vulnerability index was calculated taking into account species frequencies in the study area [20, 34, 35]. For this purpose, vegetation surveys were conducted in 152 plots distributed in different vegetation types (grass savannas, shrub savannas, tree savannas, savanna woodlands, woodland and gallery forest) and the fields throughout the study area. The sampling unit was a 50 m × 20 m (1000 m2) in non-cultivated savannas, 50 m × 10 m (500 m) in gallery forest, and 50 m × 50 m (2500 m2) in cultivated areas [36]. These plot dimensions are used to take into consideration the spatial distribution of most species with unevenly spread individuals [36].

### Data analyses

Firstly, useful species cited by informants were ranked according to the fifteen most cited ES of the four categories of provisioning, regulating, cultural and supporting ES [1, 5]. Ethnobotanical indices were calculated to assess the importance of services provided by each species. They are:

- the relative frequency of organ citation (RFO) with the adapted formula from Camou-Guerrero et al. [37] $\mathrm{RFO}= (\mathrm{Nuh }/\mathrm{ Ntu})\mathrm{ x }100$
  where Nuh represents the number of citations of uses of the organ and Ntu the number of citations of all the organs in each ES;
- the relative frequency of service citation (RFS) $\mathrm{RFS }= (\mathrm{Nuh }/\mathrm{ Ntu})\mathrm{ x }100$
  where Nuh represents the number of citations of one ES and Ntu the number of citations of all ES at one level of land use intensity.
- the actual UV index of a species (mean of the number of distinct actual uses reported per informant) $UV=\mathrm{\Sigma Ui}/Ut$

where Ui denotes the number of different uses of a species and Ut the total number of people who cited the species.

Secondly, a nonparametric test of Kruskal–Wallis at the 5% threshold was carried out to compare the different ES quoted by the populations pertaining to age classes, gender, land use intensity and sociocultural groups, where χ^2^ represents the approximate value of modal distribution. The degree of freedom, D.f = effectif-1 and a *P* value ˂ 0.05 indicate a significant difference in results. The analyses were processed with R software [38], with etnobotanyR and agricolae packages. A cluster analysis was carried out to determine the degree of similarity between the knowledge of different sociocultural groups; afterward, a principal component analysis (PCA) was performed with PcOrd9 software to assess the links between sociocultural groups (individuals) and knowledge on ES (initial variables).

Thirdly, the conservation status of the ten most used species was determined by the vulnerability index calculation [34] which is the average of the highest values of seven selected parameters (Table 2). According to Betti [34], if VI < 2, the species is assumed to be weakly vulnerable, if 2 ≤ VI < 2.5, the species is moderately vulnerable, and if VI ≥ 2.5, the species is highly vulnerable.

**Table 2:** Applied parameters of the vulnerability index

Parameters	1 (Low scale)	2 (Average scale)	3 (High scale)
Use frequency (N1)	N1 < 20%	20% ≤ N1 < 60%	N1 ≥ 60%
Number of uses (N2)	N2 < 2	2 ≤ N2 ≤ 4	N2 ≥ 5
Plant parts used (N3)	Leaves, latex	Fruits, branches	Wood, seeds, bark, roots, flowers
Biotope of plant (N4)	Ruderal, gardens, field	Secondary forest	Primary or undisturbed forest
Collection mode (N5)	Collection on the ground		Collection on the tree, cutting
Development stage (N6)	Old, senescent	Adult	Young
Relative frequency in the environment (N7)	Rf ≥ 2/3 Fm	1/3 Fm ≤ Rf < 2/3 Fm	Rf < 1/3 Fm

Rf, relative frequency; Fm, maximum frequency

An adaptation of the method of Lawrence et al. [39] was used to assess the orders of preference of causes of degradation, recommended solutions, and motivations for ecosystem preservation. The ranks given by each informant were converted into scores. The used scores were grades decreasing from the number of items in each question. For example, if 7 causes were cited for ecosystem degradation, the used scores were scores starting from 7 and decreasing in the order of the informant's citation. When an item was not cited by an informant, a score of 0 was given. For each item, the average score was calculated for each socio-cultural group, age group, and gender.

$$Vti=\sum Ti/Ni$$

Vti: the average score given to a given item by a category of informants; Ti: the sum of the scores given to this item by this category of informants; Ni: the number of informants from this category of informants.

## Results

### Diversity of used plant species and ecosystem services

**Fig. 2:**
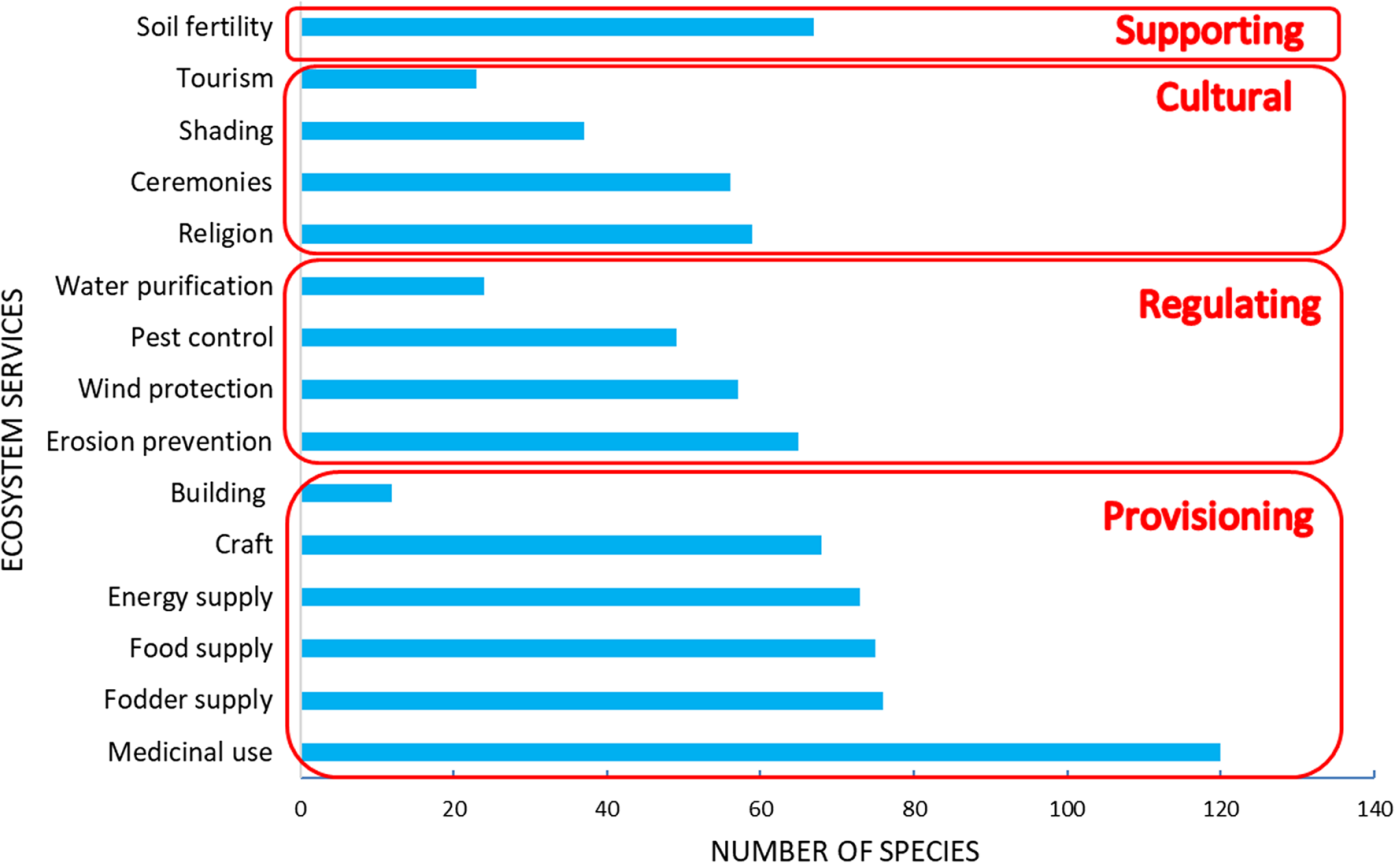
Numbers of species used for 15 ecosystem services

Major ES providers to local populations were 163 species including 130 woody species and 33 herbaceous species (belonging to 122 genera and 42 families). In all categories of land-use intensity, the most dominant families were Fabaceae (35 species), Poaceae (17 species), and Malvaceae (13 species). Each ES concerned a great diversity of plant species. At least 60 species were used for 10 ES. Provisioning services were accomplished with the highest number of species (Fig. 2) and were the most cited (67%) by the informants. These services concerned medicinal use (120 species), fodder (76 species), and food supply (75 species). The species used in these three services had multipurpose uses. *Diospyros mespiliformis*, *Khaya senegalensis*, *Vitellaria paradoxa* and *Saba senegalensis* were quoted for 14 ES.

**Fig. 3:**
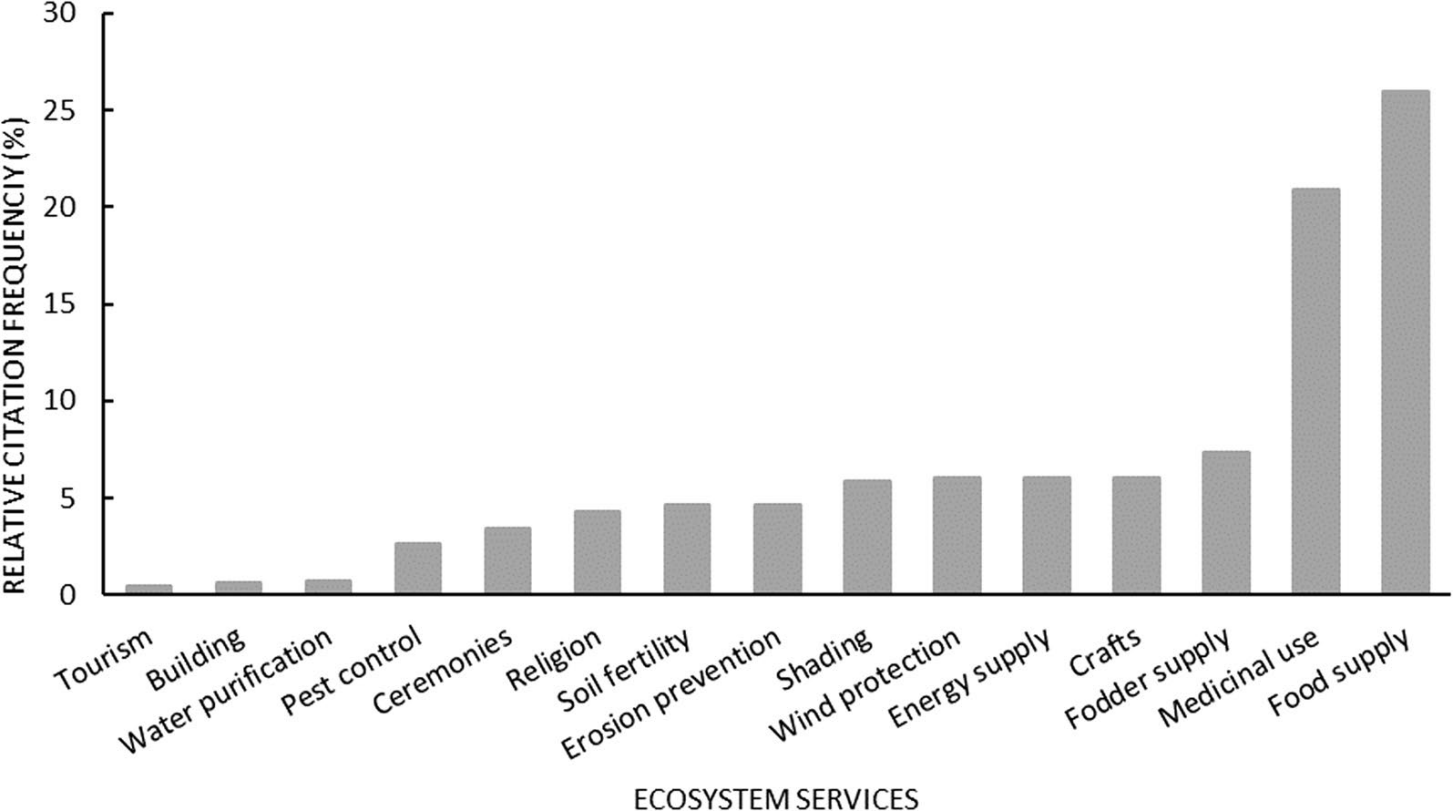
Relative frequency of citation of ecosystem services provided by plant species

When considering the study area, the relative frequencies of number of citations varied significantly between the different ES (χ^2^ = 1849.6; Dl = 14; *P* value < 0.0001). By far the most quoted ES were food supply (25.98%) and medicinal use (20.89%), followed by fodder (7.33%), craft (6.10%) and energy supply (6.09%), which are all provisioning services (Fig. 3). Protection against wind (6.08%) and shading (6%) (regulating services) were important services, too.

**Fig. 4:**
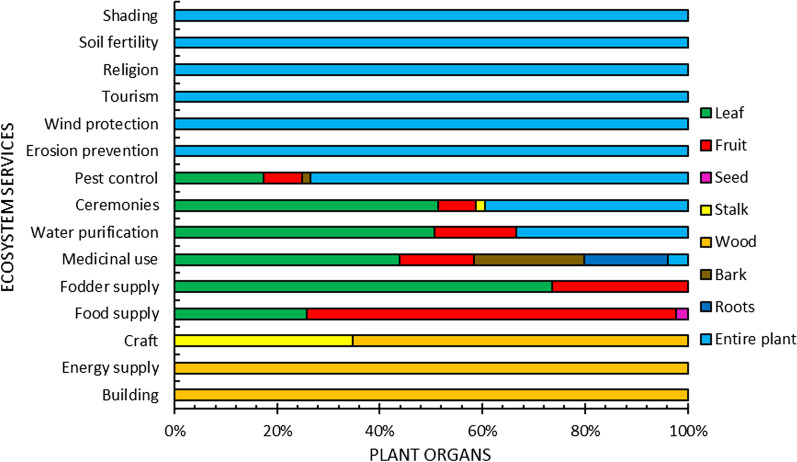
Percentage of plant organs used for ecosystem services

The relationships between ES and plant organs used for them (Fig. 4) showed that the entire plant was exclusively (100%) cited for six ES, notably soil fertility (supporting service), religion, tourism (cultural services), and shading, protection against wind and erosion prevention (regulating services).

Wood was exploited for the three provisioning services energy supply (100%), construction (100%) and craft (65%). Plant stalks were used only for craft and ceremony services. Plant leaves and fruits were cited for provisioning (food, fodder supply, medicinal use), regulating (pest control, water purification) and cultural (ceremonies) services with varying relative frequencies. Fruits were cited more often (70%) for food service. Flowers and seeds were used for food only. The highest number of organs (5) was cited for medicinal services, including bark and roots.

The use values (UV) computed for all quoted species showed that many species were exploited for several services. Woody species were the plant category with the highest UV (i.e., the most used species). *Vitellaria paradoxa* (UV = 3.775) was the species with the highest use value in the study sites. The three most used herbaceous species were *Andropogon gayanus* Kunth (UV = 0.723), *Rottboellia cochinchinensis* (Lour.) Clayton (UV = 0.400) and *Hyptis spicigera* Lam (UV = 0.370). In general, the ten species with the highest use values were in decreasing order: *Vitellaria paradoxa*, *Parkia biglobosa*, *Diospyros mespiliformis*, *Adansonia digitata*, *Lannea microcarpa, Faidherbia albida*, *Khaya senegalensis*, *Afzelia africana, Ficus sycomorus*, and *Pterocarpus erinaceus* (Table 3).

**Table 3:** Plant taxa utilized in the study area, their use values, and vulnerability indexes

Plant taxon	Plant family	Number of reported uses	Number of utilized services	Used plant parts	Use Value	Vulnerability index	Voucher number
*Acacia* spp. (ex)	Fabaceae	9	5	Lf. Wd. Ent	0.04	2.43	
*Adansonia digitata* L	Malvaceae	447	12	Lf. Fr. Se. Wd. Bk. Ent	1.693	2.71	Ouédraogo. J. 61 (OUA)
*Afzelia africana* Sm. ex Pers	Fabaceae	343	12	Lf. Fr. Wd. St. Bk. Rt. Ent	1.337	2.71	Guinko 1603 (OUA)
*Agave sisalana* Perrine [cult.]	Agavaceae	11	3	Ent	0.05	2.29	Yacouba H. 5399 (OUA)
*Anacardium occidentale* L. [cult.]	Anacardiaceae	29	8	Lf. Fr. Se. Wd. St. Bk. Ent	0.12	2.29	Thiombiano & al. 3249 (FR; OUA)
*Andropogon chinensis* (Nees) Merr	Poaceae	60	6	Lf. St. Ent	0.02	2.14	Thiombiano 319 (OUA)
*Andropogon gayanus* Kunth	Poaceae	206	8	Lf. St. Ent	0.723	2.29	Thiombiano 429 (OUA)
*Andropogon* spp.	Poaceae	18	3	Lf. St. Ent	0.08	2	
*Annona senegalensis* Pers	Anacardiaceae	74	7	Lf. Fr. Fl. Wd. Bk. Rt. Ent	0.303	2.57	Ouédraogo. J. 9 (OUA)
*Azadirachta indica* A.Juss. [cult.]	Meliaceae	307	11	Lf. Fr. Se. Fl. Wd. Bk. Rt. Ent	1.127	2.43	Kristensen 26 (OUA)
*Balanites aegyptiaca* (L.) Delile	Zygophyllaceae	175	9	Lf. Fr. Wd. Bk. Rt. Ent	0.497	2.57	Ouédraogo. J. 4 (OUA)
*Boerhavia diffusa* L	Nyctaginaceae	9	1	Ent	0.03	1.86	Thiombiano & al. 95 (OUA)
*Bombax costatum* Pellegr. & Vuill	Malvaceae	97	9	Lf. Fr. Fl. Wd. Bk. Rt. Ent	0.39	2.57	Tiné & Bambara 29 (OUA)
*Bridelia scleroneura* Müll. Arg	Phyllanthaceae	10	5	Wd. Bk. Rt. Ent	0.047	2.43	Guinko & al. 3388 (OUA)
*Burkea africana* Hook	Fabaceae	41	7	Lf. Wd. Bk. Rt. Ent	0.19	2.43	Mbayngone 335 (OUA)
*Cadaba farinosa* Forssk	Capparaceae	5	4	Lf. Rt. Ent	0.017	2.29	Ouédraogo. A. 86 (OUA)
*Calotropis procera* (Ait.) Ait. f	Apocynaceae	8	3	Lf. Fr. Rt. Ent	0.033	2.29	Guinko & al. 3423 (OUA)
*Capparis sepiaria* L	Capparaceae	11	7	Lf. Fr. Rt. Ent	0.04	2.43	Tiné & Bambara 53 (OUA)
*Capsicum frutescens* L. [cult.]	Solanaceae	9	1	Lf. Fr. Ent	0.037	2.14	Thiombianoet al. 3172 (OUA)
*Carica papaya* L. [cult.]	Caricaceae	76	3	Lf. Fr	0.283	2	N'Do 6806 (OUA)
*Cassia nigricans* Vahl	Fabaceae	62	4	Lf. Fr. Ent	0.283	2.14	Thiombiano & al. 2248 (OUA)
*Cassia obtusifolia* L	Fabaceae	13	1	Lf	0.057	1.57	Guinko 62 (OUA)
*Cassia sieberiana* DC	Fabaceae	33	4	Lf. Fr. Wd. Bk. Rt. Ent	0.107	2.29	Guinko 1340 (OUA
*Ceiba pentandra* (L.) Gaertn	Fabaceae	2	2	Ent	0.007	2.29	Kristensen 47 (OUA)
*Chasmopodium caudatum* (Hack.) Stapf	Malvaceae	64	11	Lf. Fr. Se. Wd. Bk. Ent	0.21	2.43	Tibiri A. 4390 (OUA)
*Chrysopogon nigritanus* (Benth.) Veldkamp	Poaceae	19	2	Ent	0.073	2	Mbayngone 133 (OUA)
*Citrus aurantium* L. [cult.]	Poaceae	3	1	Lf. Ent	0.01	1.86	Guinko 1023 (OUA)
*Citrus limon* (L.) Burm.f. [cult.]	Rutaceae	24	4	Lf. Fr. Se. Wd. Ent	0.107	2.14	
*Cochlospermum planchonii* Hook. f. ex Planch	Rutaceae	107	4	Lf. Fr. Se. Wd. Ent	0.387	2.14	Küppers 1293 (FR)
*Cola cordifolia* (Cav.) R.Br	Bixaceae	3	2	Fr. Se. Rt. Ent	0.013	2	Guinko 57 (OUA)
*Cola laurifolia* Mast	Malvaceae	7	1	Fr	0.033	2	Schmidt & al. 875 (OUA)
*Coldenia procumbens* L	Malvaceae	4	2	Fr	0.02	2.14	Thiombiano 210 (OUA)
*Combretum adenogonium* Steud. ex A. Rich	Boraginaceae	3	2	Lf	0.013	1.86	Guinko 612 (OUA)
*Combretum collinum* Fresen	Combretaceae	24	7	Lf. Fr. Wd. Rt. Ent	0.13	2.43	Thiombiano 729 (OUA)
*Combretum glutinosum* Perr. ex DC	Combretaceae	65	7	Lf. Fr. Wd. Bk. Rt. Ent	0.243	2.43	Guinko 1069 (OUA)
*Combretum molle* G. Don	Combretaceae	1	1	Wd	0.003	2.14	Thiombiano & al. 2442 (OUA)
Combretum nigricans Lepr. ex Guill. & Perr	Combretaceae	3	1	Lf	0.013	1.86	Thiombiano 84 (OUA)
*Combretum paniculatum* Vent	Combretaceae	1	1	Wd	0.003	1.86	Thiombiano & al. 2465 (OUA)
*Combretum sericeum* G. Don	Combretaceae	10	2	Lf. Rt. Ent	0.03	2.29	Thiombiano & al. 2003 (OUA)
*Corchorus olitorius* L	Combretaceae	7	2	Lf. Fr	0.023	2.14	Thiombiano 607 (OUA)
*Cordia myxa* L	Malvaceae	18	6	Lf. Fr. Ent	0.08	2.14	Ouoba 39 (OUA)
*Crateva adansonii* DC	Boraginaceae	180	11	Lf. Fr. Wd. St. Bk. Ent	0.75	2.57	Ouoba 36 (OUA)
*Crossopteryx febrifuga* (Afzel. ex G.Don) Benth	Capparaceae	131	8	Lf. Fr. Wd. Bk. Rt. Ent	0.537	2.57	Tiné & Bambara 52 (OUA)
*Cyanotis lanata* Benth	Rubiaceae	65	7	Lf. Fr. Wd. Bk. Rt. Ent	0.23	2.43	Madsen 5159 (OUA)
*Cymbopogon caesius* (Nees ex Hook. & Arn.) Stapf	Commelinaceae	5	1	Ent	0.023	1.86	Thiombiano 1029 (OUA)
*Cymbopogon schoenanthus* (L.) Spreng	Poaceae	34	7	Lf. St. Ent	0.113	2.14	Laegaard & al. 18306 (OUA)
*Dalbergia boehmii* Taub	Poaceae	31	7	Lf. Rt. Ent	0.143	2.14	Korbéogo 12 (OUA)
*Dalbergia melanoxylon* Guill. & Perr	Fabaceae	1	1	Wd	0.003	2.14	Ouattara 75 (OUA)
*Daniellia oliveri* (Rolfe) Hutch. & Dalziel	Fabaceae	94	10	Lf. Wd. St. Bk. Rt. Ent	0.36	2.57	Ouédraogo. A. 17 (OUA)
*Detarium microcarpum* Guill. & Perr	Fabaceae	338	13	Lf. Fr. Se. Wd. Bk. Rt. Ent	1.21	2.57	Thiombiano 808 (OUA)
*Dichrostachys cinerea* (L.) Wight & Arn	Fabaceae	14	7	Lf. Wd. Ent	0.047	2.43	Ouoba 2001 (OUA)
*Dicoma tomentosa* Cass	Asteraceae	1	1	Ent	0.003	1.86	Madsen 5926 (OUA)
*Diospyros mespiliformis* Hochst. ex A. DC	Ebenaceae	402	14	Lf. Fr. Se. Wd. St. Bk. Rt. Ent	2.249	2.71	Ouédraogo. J. 35 (OUA)
*Echinochloa pyramidalis* (Lam.) Hitchc. & Chase	Poaceae	23	3	St. Ent	0.077	2	Laegaard 21295 (OUA)
*Entada africana Guill*. & Perr	Fabaceae	32	5	Wd. Bk. Ent	0.15	2.43	Guinko 733 (OUA)
*Erythrina senegalensis* A.DC	Fabaceae	4	3	Lf. Bk. Ent	0.02	2.29	Guinko 116 (OUA)
*Eucalyptus camaldulensis* Dehnh. [cult.]	Myrtaceae	78	9	Lf. Fr. Wd. Bk. Rt. Ent	0.32	2.29	Rosendal 6876 (OUA)
*Fadogia agrestis* Schweinf. ex Hiern	Rubiaceae	2	2	Lf. Ent	0.007	2.29	Thiombiano & al. 21 (OUA)
*Faidherbia albida* (Delile) A. Chev. Del	Fabaceae	384	11	Lf. Fr. Wd. Bk. Rt. Ent	1.53	2.43	Tibiri A. 4369 (OUA)
*Feretia apodanthera* Delile	Rubiaceae	20	5	Lf. Wd. Bk. Rt. Ent	0.083	2.43	Guinko 1370 (OUA)
*Ficus abutilifolia* (Miq.) Miq	Moraceae	12	4	Lf. Fr.Wd. Ent	0.037	2.29	Thiombiano 238 (OUA)
*Ficus dicranostyla* Mildbr	Moraceae	9	2	Lf. Fr. Bk	0.037	2.29	Schmidt & al. 913 (OUA)
*Ficus ingens* (Miq.) Miq	Moraceae	1	1	Fr	0.033	2	Ouédraogo. O. 104 (OUA)
*Ficus platyphylla* Delile	Moraceae	22	7	Lf. Fr. Wd. Bk. Ent	0.083	2.43	Guinko & al. 6004 (OUA)
*Ficus sur* Forssk	Moraceae	6	3	Fr. Bk. Ent	0.02	2.29	Ouoba 19 (OUA)
*Ficus sycomorus* L	Moraceae	337	12	Lf. Fr. Wd. St. Bk. Rt. Ent	1.273	2.57	Ouédraogo. A. 133 (OUA)
*Flacourtia indica* (Burm. f.) Merrill	Salicaceae	4	2	Lf. Fr	0.02	2.14	Ouoba 18 (OUA)
*Flueggea virosa* (Roxb. ex Willd.) Voigt	Phyllanthaceae	6	2	Lf. Wd. St	0.02	2.29	Thiombiano 226 (OUA)
*Gardenia erubescens* Stapf & Hutch	Rubiaceae	196	10	Lf. Fr. Wd. Bk. Rt. Ent	0.857	2.57	Guinko 374 (OUA)
*Gardenia ternifolia* Schumach. & Thonn	Rubiaceae	3	2	Fr. Rt. Ent	0.013	2.29	Thiombiano & al. 285 (OUA)
*Gmelina arborea* Roxb. [cult.]	Lamiaceae	16	6	Lf. Fr. Fl. Ent	0.07	2.43	Thiombiano & al. 43 (OUA)
*Grewia bicolor* Juss	Malvaceae	2	1	Fr	0.007	2	Thiombiano & al. 407 (OUA)
*Grewia cissoides* Hutch. & Dalziel	Malvaceae	1	1	Fr	0.003	2	Thiombiano & al. 191 (OUA)
*Grewia lasiodiscus* K.Schum	Malvaceae	8	2	Lf. Fr. Wd	0.037	2.29	Thiombiano 614 (OUA)
*Grewia* spp	Malvaceae	3	2	Ent	0.01	2.29	
*Guiera senegalensis* J.F.Gmel	Combretaceae	24	2	Lf. St. Ent	0.08	2.29	Ouédraogo. A. 239 (OUA)
*Gymnosporia senegalensis* (Lam.) Loes	Celastraceae	21	6	Lf. Fl. Wd. Bk. Rt. Ent	0.083	2.29	Schmidt & al. 912 (OUA)
*Haematostaphis barteri* Hook.f	Anacardiaceae	4	1	Lf	0.013	1.86	Ouédraogo. A. 102 (OUA)
*Hibiscus cannabinus* L. [cult.]	Malvaceae	12	3	Lf. Fr. Se. Fl. Ent	0.037	2	Ouédraogo. O. 364 (OUA)
*Hymenocardia acida* Tul	Phyllanthaceae	9	2	Lf. Wd	0.03	2.29	Guinko 888 (OUA)
*Hyparrhenia* spp	Poaceae	2	2	Ent	0.007	2	Ouoba 157 (OUA); Madsen 6003 (OUA); Mbayngone 125 (OUA)
*Hyptis spicigera* Lam	Lamiaceae	86	6	Lf. Ent	0.37	2.29	Guinko 610 (OUA)
*Indigofera bracteolata* DC	Fabaceae	2	2	Rt. Ent	0.01	2	Ouattara 71 (OUA)
*Ipomoea carnea* Jacq	Convolvulaceae	3	1	Lf. Ent	0.007	2	Guinko 1290 (OUA)
*Isoberlinia doka* Craib & Stapf	Fabaceae	69	10	Lf. Fr. Wd. Bk. Ent	0.26	2.57	Thiombiano & al. 319 (OUA);
*Jatropha curcas* L. [cult.]	Euphorbiaceae	31	8	Ent	0.123	2.29	Tibiri A. 4403 (OUA)
*Khaya senegalensis* (Desv.) A. Juss	Meliaceae	403	14	Lf. Fr. Wd. Bk. Rt. Ent	1.503	2.71	Sawadogo 6883 (OUA)
*Kigelia africana* (Lam.) Benth	Bignoniaceae	19	4	Lf. Fr. Bk. Rt. Ent	0.077	2.29	Thiombiano 469 (OUA)
*Landolphia heudelotii* A. DC	Apocynaceae	13	3	Lf. Fr. St	0.05	2.29	Guinko & al. 5085 (OUA)
Lannea acida A. Rich	Anacardiaceae	55	10	Lf. Fr. Wd. St. Bk. Rt. Ent	0.207	2.29	Ouédraogo. J. 87 (OUA)
*Lannea microcarpa* Engl. & K. Krause	Anacardiaceae	371	13	Lf. Fr. Wd. Bk. Rt. Ent	1.583	2.71	Guinko 1383 (OUA)
*Lannea velutina* A.Rich	Anacardiaceae	1	1	Bk	0.003	2.14	Ouédraogo. A. 105 (OUA)
*Leptadenia hastata* (Pers.) Decne	Apocynaceae	14	4	Lf. Fr. Fl. Rt. Ent	0.06	2.29	Thiombiano & al. 2028 (OUA)
*Loeseneriella africana* (Willd.) N.Hallé	Celastraceae	1	1	Lf	0.003	1.86	Ouédraogo. O. 67 (OUA)
*Lophira lanceolata* Tiegh. ex Keay	Ochnaceae	78	5	Lf. Fr. Wd. Bk. Rt. Ent	0.337	2.43	Guinko 2063 (OUA)
*Loudetia simplex* (Nees) C.E.Hubb	Poaceae	2	2	Lf. St	0.01	1.86	Laegaard & al. 21140 (OUA)
*Loudetia togoensis* (Pilg.) C.E.Hubb	Poaceae	1	1	Ent	0.003	1.86	Mbayngone 45 (OUA)
*Maerua angolensis* DC	Capparaceae	7	2	Lf. Fl. Wd	0.023	2.43	Ouédraogo. A. 20 (OUA)
*Maerua crassifolia* Forssk	Capparaceae	3	2	Lf. Ent	0.013	2.29	Guinko 2350 (OUA)
*Mangifera indica* L. [cult.]	Anacardiaceae	359	13	Lf. Fr. Wd. Bk. Rt. Ent	1.617	2.43	Ouédraogo. H. 35 (OUA)
*Mimosa pigra* L	Fabaceae	5	2	Wd. Ent	0.017	2.29	Guinko 605 (OUA)
*Mitragyna inermis* (Willd.) Kuntze	Rubiaceae	53	9	Lf. Fr. Wd. St. Bk. Rt. Ent	0.433	2.43	Schmidt & al. 6369 (OUA)
*Moringa oleifera* L	Moringaceae	157	8	Lf. Fr. Wd. Bk. Rt. Ent	0.6	2.43	Thiombiano & al. 3877 (OUA)
*Nymphaea lotus* L	Nymphaeaceae	8	4	Lf. Rt. Ent	0.033	2.29	Madsen 5649 (OUA)
*Ocimum americanum* L	Lamiaceae	48	5	Lf. Ent	0.197	2.14	Guinko & al. 3489 (OUA)
*Oncoba spinosa* Forssk	Salicaceae	32	4	Lf. Fr. Wd. Bk. Rt. Ent	0.133	2.29	Thiombiano & Boussim 283 (OUA);
*Opilia amentacea* Roxb	Opiliaceae	20	3	Lf. Fr. Rt. Ent	0.067	2.29	Ouédraogo. J. 82 (OUA)
*Oxytenanthera abyssinica* (A.Rich.) Munro	Poaceae	1	1	St	0.003	2	Schmidt & al. 893 (OUA)
*Ozoroa obovata* (Oliv.) R.Fern. & A.Fern	Anacardiaceae	1	1	Lf	0.003	1.86	Thiombiano & al. 2449 (OUA)
*Parinari curatellifolia* Planch. ex Benth	Chrysobalanaceae	7	3	Lf. Fr. Bk. Ent	0.027	2.29	Ouédraogo. A. 170 (OUA)
*Parkia biglobosa* (Jacq.) R. Br. ex G. Don f	Fabaceae	584	12	Lf. Fr. Se. Wd. Bk. Rt. Ent	2.353	2.57	Madsen 5113 (OUA)
*Paullinia pinnata* L	Sapindaceae	8	1	Lf. Rt	0.023	2.14	Guinko 604 (OUA)
*Pennisetum pedicellatum* Trin	Poaceae	8	5	Lf. St. Ent	0.037	2.14	Thiombiano 1007 (OUA)
*Pennisetum* spp	Poaceae	29	3	St. Ent	0.107	2	Thiombiano 1007 (OUA) Laegaard & al. 18412 (OUA)
*Pericopsis laxiflora* (Benth. ex Bak.) van Meeuwen	Fabaceae	23	6	Lf. Wd. Rt. Ent	0.103	2.43	Thiombiano 853 (OUA)
*Philenoptera laxiflora* (Guill. & Perr.) Roberty	Fabaceae	29	8	Lf. Fr. Wd. Bk. Rt. Ent	0.117	2.43	Taïta 203 (OUA)
*Piliostigma reticulatum* (DC.) Hochst	Fabaceae	30	8	Lf. Fr. Wd. St. Bk. Ent	0.113	2.43	Thiombiano 604 (OUA)
*Piliostigma thonningii* (Schum.) Milne-Redhead	Fabaceae	135	11	Lf. Fr. Wd. St. Bk. Rt. Ent	0.563	2.43	Thiombiano & al. 2200 (OUA)
*Prosopis africana* (Guill. & Perr.) Taub	Fabaceae	31	7	Lf. Fr. Wd. Bk. Rt. Ent	0.13	2.43	Kristensen 44 (OUA)
*Pseudocedrela kotschyi* (Schweinf.) Harms	Meliaceae	36	9	Lf. Wd. Bk. Ent	0.13	2.43	Thiombiano 194 (OUA)
*Psidium guajava* L. [cult.]	Myrtaceae	43	4	Lf. Fr. Wd. Bk. Rt. Ent	0.193	2.14	Ouédraogo. H. 40 (OUA)
*Pterocarpus erinaceus* Poir	Fabaceae	297	13	Lf. Fr. Wd. Bk. Rt. Ent	1.237	2.71	Guinko 1030 (OUA)
*Pterocarpus santalinoides* DC	Fabaceae	2	2	Se. Wd	0.007	2.29	Ouédraogo. O. 177 (OUA)
*Raphionacme splendens* Schltr	Apocynaceae	2	2	Rt. Ent	0.01	2.29	Thiombiano & al. 2833 (OUA)
*Rottboellia cochinchinensis* (Lour.) Clayton	Poaceae	85	8	Lf. Ent	0.4	2.29	Guinko 1890 (OUA)
*Rytigynia senegalensis* Blume	Rubiaceae	1	1	Wd	0.003	2.14	Schmidt & al. 1189 (FR; OUA)
*Saba senegalensis* (A. DC.) Pichon	Apocynaceae	239	14	Lf. Fr. Se. Wd. St. Bk. Rt. Ent	1.027	2.57	Guinko & al. 6000 (OUA)
*Sarcocephalus latifolius* (Sm.) E.A.Bruce	Rubiaceae	65	5	Lf. Fr. Wd. Bk. Rt	0.253	2.43	Guinko 661 (OUA)
*Sclerocarya birrea* (A. Rich.) Hochst	Anacardiaceae	31	8	Lf. Fr. Wd. St. Bk. Ent	0.12	2.43	Ouédraogo. J. 15 (OUA)
*Securidaca longipedunculata* Fresen	Polygalaceae	52	6	Lf. Fr. Wd. Bk. Rt. Ent	0.21	2.43	Guinko 392 (OUA)
*Senegalia ataxacantha* (DC.) Kyal. & Boatwr	Fabaceae	12	8	Lf. Fr. Wd. Bk. Ent	0.027	2.43	Tiné & Bambara 25 (OUA)
*Senegalia gourmaensis* (A. Chev.) Kyal. & Boatwr	Fabaceae	33	7	Lf. Fr. Wd. Bk. Ent	0.107	2.43	Ouédraogo. A. 80 (OUA)
*Senegalia macrostachya* (Reichenb. ex DC.) Kyal. & Boatwr	Fabaceae	12	5	Lf. Fr. Wd. Ent	0.05	2.43	Madsen 5528 (OUA)
*Senegalia polyacantha* (Willd.) Seigler & Ebinger	Fabaceae	14	4	Lf. Fr. Bk. Rt. Ent	0.04	2.29	Ouédraogo. A. 135 (OUA)
*Senegalia senegal* (L.) Britton	Fabaceae	1	1	Lf	0.333	1.86	Ouédraogo. A. 136 (OUA)
*Sporobolus pyramidalis* P.Beauv	Poaceae	7	3	St. Ent	0.017	2	Martinussen 55 (OUA)
*Sterculia setigera* Delile	Malvaceae	53	6	Lf. Fr. Se. Bk. Rt. Ent	0.217	2.43	Thiombiano 858 (OUA)
*Stereospermum kunthianum* Cham	Bignoniaceae	7	1	Ent	0.023	2.14	Ouédraogo. J. 57 (OUA);
*Striga hermonthica* (Delile) Benth	Orobanchaceae	51	5	Lf. Ent	0.243	2.43	Guinko 2323 (OUA)
*Strophanthus hispidus* DC	Apocynaceae	13	4	Lf. Fr. Rt. Ent	0.053	2.43	Thiombiano & al. 2693 (OUA)
*Strychnos innocua* Delile	Loganiaceae	5	3	Lf. Rt. Ent	0.019	2.29	Guinko 114 (OUA);
*Strychnos spinosa* Lam	Loganiaceae	99	8	Lf. Fr. Wd. Ent	0.277	2.57	Guinko 1360 (OUA)
*Tamarindus indica* L	Fabaceae	332	13	Lf. Fr. Se. Wd. Bk. Rt. Ent	0.99	2.57	Thiombiano 1063 (OUA)
*Tapinanthus* spp.	Loranthaceae	27	6	Lf. Fr. Wd. Ent	0.117	2.29	Boussim 10 (OUA); Boussim 15 (OUA); Boussim 16 (OUA)
*Tectona grandis* L.f. [cult.]	Lamiaceae	112	9	Lf. Fl. Wd.. Ent	0.533	2.57	Schmidt & al. 1086 (OUA)
*Tephrosia linearis* (Willd.) Pers	Fabaceae	3	3	Lf. Rt. Ent	0.013	2	Thiombiano 956 (OUA)
*Terminalia avicennioides* Guill. & Perr	Combretaceae	45	9	Lf. Wd. Bk. Rt. Ent	0.16	2.43	Thiombiano 613 (OUA); Thiombiano 229 (OUA);
*Terminalia engleri* Gere & Boatwr.,	Combretaceae	19	5	Lf. Wd. Bk. Rt. Ent	0.06	2.43	Taïta 209 (OUA)
*Terminalia macroptera* Guill. & Perr	Combretaceae	78	12	Lf. Fr. Wd. Bk. Rt. Ent	0.307	2.43	Thiombiano 905 (OUA)
*Terminalia schimperi* Hochst. ex Hutch. & Dalziel	Combretaceae	148	12	Lf. Fr. Wd. Bk. Rt. Ent	0.593	2.57	Schmidt & al. 1186 (FR; OUA)
*Trichilia emetica* Vahl	Meliaceae	19	8	Lf. Fr. St. Bk. Rt. Ent	0.067	2.43	Guinko 117 (OUA)
*Triumfetta lepidota* K.Schum	Malvaceae	13	2	Ent	0.043	2.14	Guinko 1052 (OUA)
*Vachellia nilotica* (L.) P.J.H.Hurter & Mabb	Fabaceae	66	8	Lf. Fr. Bk. Rt. Ent	0.303	2.43	Thiombiano 3080 (OUA)
*Vachellia seyal* (Del.) P.J.H.Hurter	Fabaceae	3	2	Lf. Rt	0.01	2.29	Guinko 851 (OUA)
*Vachellia sieberiana* (DC.) Kyal. & Boatwr	Fabaceae	16	8	Lf. Wd. Rt. Ent	0.056	2.43	Madsen 5111 (OUA)
*Vernonia colorata* (Willd.) Drake	Asteraceae	25	6	Lf. Fr. Wd. Ent	0.11	2.29	Thiombiano & al. 229 (OUA)
*Vitellaria paradoxa* C.F. Gaertn	Sapotaceae	960	14	Lf. Fr. Se. Wd. St. Bk. Rt. Ent	3.775	2.43	Madsen 5171 (OUA)
*Vitex chrysocarpa* Planch. ex Benth	Lamiaceae	9	2	Lf. Fr	0.083	2.14	Taïta 5 (OUA)
*Vitex doniana* Sweet	Lamiaceae	189	9	Lf. Fr. Wd. Bk. Rt. Ent	0.54	2.57	Ouoba 175 (OUA)
*Ximenia americana* L	Ximeniaceae	165	5	Lf. Fr. Wd. Bk. Rt. Ent	0.653	2.57	Guinko 1163 (OUA)
*Zanthoxylum zanthoxyloides* (Lam.) Zepernick & Timler	Rutaceae	13	4	Lf. Fr. Bk. Rt. Ent	0.057	2.29	Ouoba 48 (OUA)
*Ziziphus mauritiana* Lam	Rhamnaceae	19	3	Lf. Rt. Ent	0.072	2.29	Thiombiano & al. 199 (OUA)
*Ziziphus mucronata* Willd	Rhamnaceae	3	2	Lf. Rt	0.01	2.29	Guinko & al. 3373 (OUA)

Used plant parts: Lf. = Leaf; Fl. = Flower; Fr. = Fruit; Se. = Seed; Wd. = Wood; St. = stalk; Bk. = Bark; Rt. = Root; Ent = Entire plant

### Variables influencing local knowledge of ecosystem services

With regard to the knowledge of local populations in different land-use intensity areas (Table 4), the citation of ES varied significantly (*p* < 0.05), except for soil fertility, water purification and pest control services (*p* > 0.05). With regard to the sociocultural groups (Table 4), the citation of ES varied significantly (*p* < 0.05) except for pest control services (*p* > 0.05). With regard to the gender of the informant, the citation of ES varied significantly (*p* < 0.05) only for food supply, fodder supply, crafts and soil fertility services. With regard to age classes, the citation of ES varied significantly (*p* < 0.05) only for medicinal use. The two variables associated with most differentiated citation of ES are the land use intensity levels and the sociocultural groups.

**Table 4:** Variation in citation of ecosystem services provided by the ten most used species across the three land use intensity levels, the sociocultural groups, the gender, and age classes

ES categories	ES	land use intensity level			Sociocultural groups			Gender			Age classes		
		D.f	χ^2^	*P* value	D.f	χ^2^	*P* value	D.f	χ^2^	*P* value	D.f	χ^2^	*P* value
Supporting	Soil fertility	2	5.77	0.06	3	15.35	0.001537	1	14.59	0.0001	2	3.19	0.20
Cultural	Tourism	2	47.11	< 0.0001	3	50.25	< 0.0001	1	1.02	0.31	2	3.37	0.18
	Religion	2	60.16	< 0.0001	3	101.25	< 0.0001	1	0.14	0.71	2	0.43	0.81
	Ceremonies	2	36.61	< 0.0001	3	76.85	< 0.0001	1	0.19	0.66	2	0.87	0.65
Regulating	Water purification	2	1.59	0.45	3	22.41	< 0.0001	1	1.48	0.22	2	3.49	0.17
	Shading	2	50.37	< 0.0001	3	51.98	< 0.0001	1	0.22	0.64	2	1.30	0.52
	Wind protection	2	42.20	< 0.0001	3	58.24	< 0.0001	1	0.34	0.56	2	0.19	0.91
	Pest control	2	4.14	0.13	3	5.00	0.1716	1	12.52	0.0004	2	0.70	0.70
	Erosion prevention	2	33.33	< 0.0001	3	31.59	< 0.0001	1	0.53	0.47	2	5.45	0.06
Provisioning	Medicinal use	2	126.51	< 0.0001	3	142.52	< 0.0001	1	0.66	0.41	2	6.03	0.048
	Construction	2	44.13	< 0.0001	3	52.04	< 0.0001	1	0.07	0.79	2	4.78	0.09
	Crafts	2	61.24	< 0.0001	3	38.48	< 0.0001	1	6.29	0.01	2	5.33	0.07
	Energy supply	2	14.38	0.0007	3	6.89	< 0.0001	1	0.38	0.53	2	0.50	0.78
	Fodder supply	2	36.22	< 0.0001	3	24.11	< 0.0001	1	4.68	0.03	2	2.21	0.33
	Food supply	2	61.18	< 0.0001	3	85.72	< 0.0001	1	7.26	0.007	2	5.82	0.054

ES, ecosystem services

**Fig. 5:**
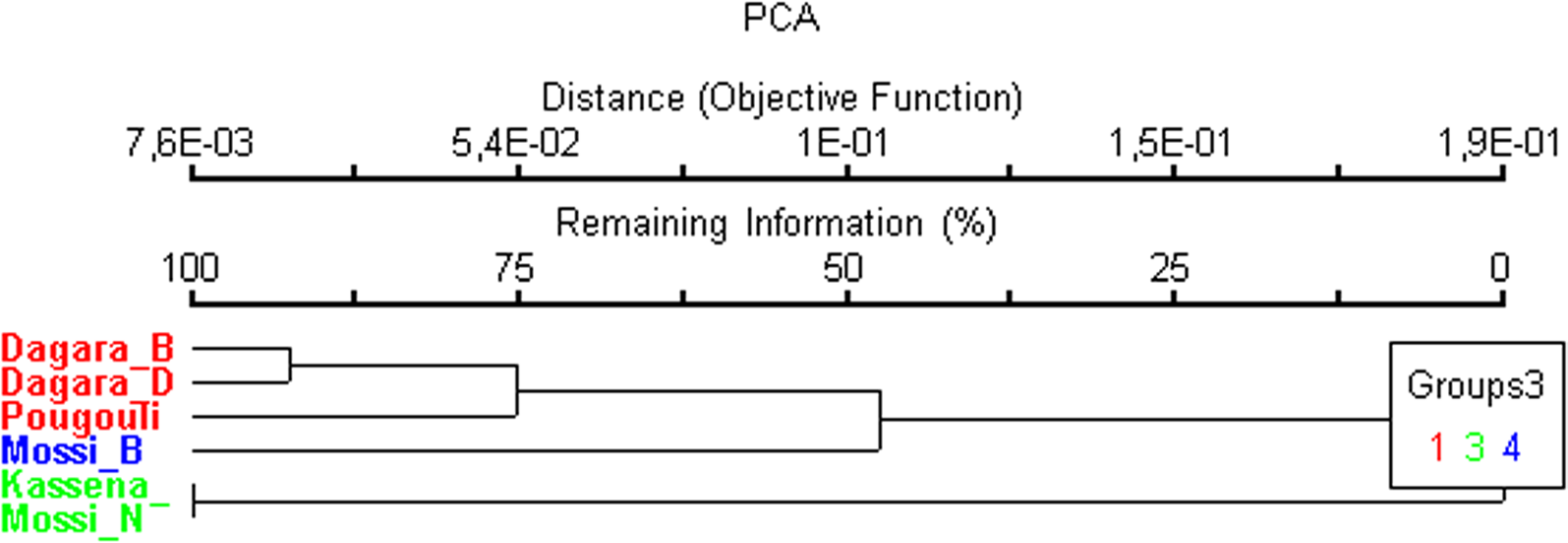
Sociocultural group scores clustered according to similarity of citation of ecosystem services. Dagara_B: Dagara of TWRB (native); Dagara_D: Dagara of CAD (native); Mossi_N: Mossi of GRN (immigrant); Mossi_B: Mossi of TWRB (immigrant)

In order to understand a combined effect of land use area and sociocultural group, six sociocultural groups at the three land use sites were subjected to a cluster analysis, based on the citations of utilized ES (Fig. 5). These are: the groups of GRN Kassena, GRN Mossi, TWRB Mossi, CAD Pougouli, CAD Dagara and TWRB Dagara. The cluster analysis discriminated three groups, one comprising the Kassena and the Mossi at GRN, which is very different from the other two being composed of the natives at TWRB and CAD (the Dagara and the Pougouli) on the one hand and the Mossi at TWRB on the other hand.

**Fig. 6:**
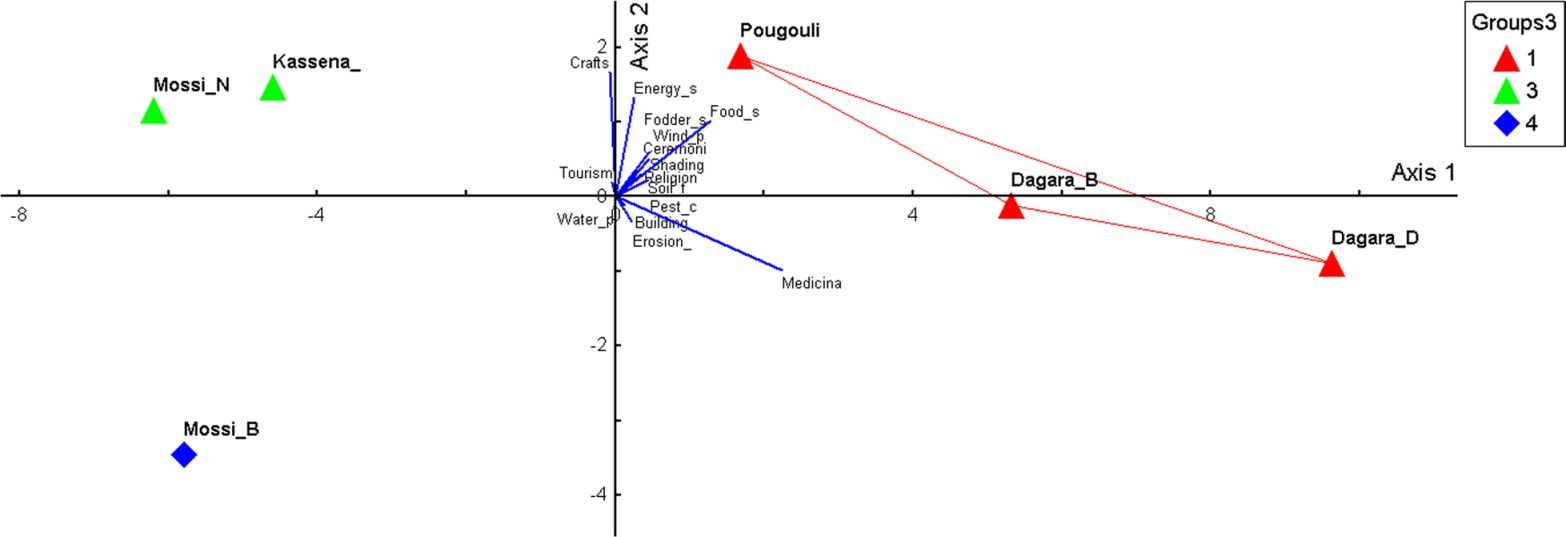
PCA of sociocultural groups and knowledge on ecosystem services. Soil_f: soil fertility; Pest_c: pest control; Erosion_: erosion prevention; Fodder_s: fodder supply; Medicina: medicinal use; Food_s: food supply; Energy_s: energy supply; Wind_p: wind protection; Water_p: water purification; Dagara_B: Dagara of TWRB; Dagara_D: Dagara of CAD; Mossi_N: Mossi of GRN; Mossi_B: Mossi of TWRB

Using the same data matrix, a principal component analysis grouped the 15 ES (initial variables) into 5 synthetic variables or principal components. The two first principal components are represented by the graph of the PCA ordination (Fig. 6). The first component (Axis 1) explains 88.08% of the total variation, the second component (axis 2) 8.04%. Therefore, these two axes explaining 96.12% of the total variation were used to describe relationships between sociocultural groups at different sites and ES. Axis 1 discriminated the natives of CAD and TWRB from the immigrant Mossi and the Kassena native to GRN. According to this axis, the Mossi of TWRB make use of the 15 ES only to a small degree and GRN residents mainly use the craft and tourism service while the natives of CAD and TWRB utilize the majority of services. Axis 2 discriminates ES utilization by the Mossi of TWRB from that by the GRN residents. Axis 2 underlines that the Mossi of TWRB make use of the 15 ES only to a small degree. The principal component analysis indicates that the utilization of ES is a function of the level of land use intensity and the economic benefits that people derive from plant formations. Thus, the GRN populations utilize the craft services related to tourism as they benefit from the financial income from tourism and participate in the management of the ranch. However, the natives of TWRB who do not benefit financially from the wildlife reserve of Bontioli and the natives of the anthropogenically shaped CAD utilize the ES that sustain the quality of the agricultural land and meet the primary needs of the rural populations.

### Vulnerability of the species

The vulnerability indices of the used species in the study area varied from 1.57 to 2.71 (Table 3), indicating varying utilization pressures by local populations. *Adansonia digitata, Afzelia africana, Diospyros mespiliformis, Khaya senegalensis, Lannea microcarpa* and *Pterocarpus erinaceus* were most vulnerable with IV = 2.71. Eighteen other species were highly vulnerable with IV > 2.5 (Table 3). The ten most utilized species were highly vulnerable, except for *Faidherbia albida* and *Vitellaria paradoxa* classified as moderately vulnerable (2 ˂ IV ≤ 2.5).

### Local perceptions of availability and population dynamics of utilized species

The relative frequency of citation regarding the availability of all utilized species (Table 5) revealed that 60.12% of citations mentioned the abundant (45.86%) to very abundant (14.24%) of all utilized species. Among the most used species, *Vitellaria paradoxa* was most cited as very abundant species (35.04%). In contrast, *Adansonia digitata* was most cited as rare species (58.05%) followed by *Faidherbia albida* (55.20%) which is only found in agrosystems.

**Table 5:** Relative frequency of perception of availability and dynamics of species utilized by local populations

Species	Species availability (%)			Species dynamics (%)		
	Rare	Abundant	Very abundant	Decrease	Constancy	Increase
All species	39.88	45.86	14.24	40.08	19.05	40.86
*Acacia* spp (ex)	50.00	50.00	0.00	33.33	50.00	16.67
*Adansonia digitata* L	58.05	29.89	12.07	52.30	19.54	28.16
*Afzelia africana* Sm. ex Pers	38.25	54.10	7.65	40.44	17.49	42.08
*Agave sisalana* Perrine [cult.]	45.45	36.36	18.18	36.36	18.18	45.45
*Anacardium occidentale* L. [cult.]	53.33	26.67	20.00	33.33	26.67	40.00
*Andropogon chinensis* (Nees) Merr	4.35	71.74	23.91	15.22	2.17	82.61
*Andropogon gayanus* Kunth	10.53	37.72	51.75	7.89	6.14	85.96
*Andropogon* spp	52.94	29.41	17.65	11.76	47.06	41.18
*Annona senegalensis* Pers	37.50	52.08	10.42	45.83	12.50	41.67
*Azadirachta indica* A.Juss. [cult.]	50.48	43.81	5.71	36.19	23.81	40.00
*Balanites aegyptiaca* (L.) Delile	39.51	54.32	6.17	46.91	16.05	37.04
*Boerhavia diffusa* L	22.22	77.78	0.00	44.44	11.11	44.44
*Bombax costatum* Pellegr. & Vuill	60.00	35.38	4.62	75.38	4.62	20.00
*Bridelia scleroneura* Müll. Arg	100.00	0.00	0.00	71.43	0.00	28.57
*Burkea africana* Hook	53.57	35.71	7.14	42.86	25.00	28.57
*Cadaba farinosa* Forssk	0.00	100.00	0.00	50.00	0.00	50.00
*Calotropis procera* (Ait.) Ait. f	37.50	62.50	0.00	50.00	12.50	37.50
*Capparis sepiaria* L	66.67	11.11	22.22	66.67	0.00	33.33
*Capsicum frutescens* L. [cult.]	0.00	57.14	42.86	0.00	0.00	100.00
*Carica papaya* L. [cult.]	84.62	12.82	2.56	56.41	17.95	25.64
*Cassia nigricans* Vahl	49.09	18.18	32.73	34.55	5.45	60.00
*Cassia obtusifolia* L	23.08	38.46	38.46	7.69	7.69	84.62
Cassia sieberiana DC	60.00	40.00	0.00	68.00	20.00	12.00
*Ceiba pentandra* (L.) Gaertn	71.43	25.00	3.57	50.00	21.43	28.57
*Chasmopodium caudatum* (Hack.) Stapf	22.22	44.44	33.33	33.33	5.56	61.11
*Chrysopogon nigritanus* (Benth.) Veldkamp	100.00	0.00	0.00	100.00	0.00	0.00
*Citrus aurantium* L. [cult.]	41.18	47.06	11.76	29.41	35.29	35.29
*Citrus limon* (L.) Burm.f. [cult.]	73.81	23.81	2.38	38.10	28.57	33.33
*Cochlospermum planchonii* Hook. f. ex Planch	66.67	33.33	0.00	66.67	33.33	0.00
*Cola cordifolia* (Cav.) R.Br	42.86	57.14	0.00	42.86	57.14	0.00
*Cola laurifolia* Mast	33.33	33.33	33.33	33.33	0.00	66.67
*Coldenia procumbens* L	50.00	50.00	0.00	0.00	0.00	100.00
*Combretum adenogonium* Steud. ex A. Rich	47.62	52.38	0.00	47.62	28.57	23.81
*Combretum collinum* Fresen	45.65	47.83	6.52	43.48	39.13	17.39
Combretum glutinosum Perr. ex DC	0.00	100.00	0.00	100.00	0.00	0.00
*Combretum molle* G. Don	66.67	33.33	0.00	66.67	0.00	33.33
*Combretum nigricans* Lepr. ex Guill. & Perr	100.00	0.00	0.00	100.00	0.00	0.00
*Combretum paniculatum* Vent	62.50	37.50	0.00	62.50	0.00	37.50
*Combretum sericeum* G. Don	40.00	60.00	0.00	80.00	0.00	20.00
*Corchorus olitorius* L	29.41	52.94	17.65	35.29	5.88	58.82
*Cordia myxa* L	40.26	53.25	6.49	33.77	37.66	28.57
*Crateva adansonii* DC	63.64	24.68	11.69	46.75	19.48	33.77
*Crossopteryx febrifuga* (Afzel. ex G.Don) Benth	30.61	67.35	2.04	53.06	16.33	30.61
*Cyanotis lanata* Benth	80.00	20.00	0.00	0.00	40.00	60.00
*Cymbopogon caesius* (Nees ex Hook. & Arn.) Stapf	10.71	50.00	39.29	25.00	0.00	75.00
*Cymbopogon schoenanthus* (L.) Spreng	30.43	43.48	26.09	52.17	13.04	34.78
*Dalbergia boehmii* Taub	0.00	100.00	0.00	0.00	0.00	100.00
*Dalbergia melanoxylon* Guill. & Perr	0.00	100.00	0.00	100.00	0.00	0.00
*Daniellia oliveri* (Rolfe) Hutch. & Dalziel	26.15	66.15	7.69	35.38	20.00	46.15
*Detarium microcarpum* Guill. & Perr	32.03	54.90	13.07	35.95	18.95	45.10
*Dichrostachys cinerea* (L.) Wight & Arn	41.67	50.00	8.33	58.33	16.67	25.00
*Dicoma tomentosa* Cass	0.00	0.00	100.00	0.00	0.00	100.00
*Diospyros mespiliformis* Hochst. ex A. DC	23.70	65.90	10.40	38.15	23.12	38.73
*Echinochloa pyramidalis* (Lam.) Hitchc. & Chase	11.11	77.78	11.11	27.78	0.00	72.22
*Entada africana* Guill. & Perr	64.29	32.14	3.57	28.57	21.43	50.00
*Erythrina senegalensis* A.DC	50.00	50.00	0.00	25.00	25.00	50.00
*Eucalyptus camaldulensis* Dehnh. [cult.]	63.64	30.30	6.06	33.33	18.18	48.48
*Fadogia agrestis* Schweinf. ex Hiern	0.00	100.00	0.00	0.00	0.00	100.00
*Faidherbia albida* (Delile) A.Chev. Del	55.20	39.20	5.60	45.60	24.80	29.60
*Feretia apodanthera* Delile	22.22	66.67	11.11	22.22	33.33	44.44
*Ficus abutilifolia* (Miq.) Miq	0.00	77.78	22.22	33.33	22.22	44.44
*Ficus dicranostyla* Mildbr	87.50	0.00	12.50	100.00	0.00	0.00
*Ficus ingens* (Miq.) Miq	0.00	100.00	0.00	100.00	0.00	0.00
*Ficus platyphylla* Delile	41.67	50.00	8.33	33.33	16.67	50.00
*Ficus sur* Forssk	50.00	50.00	0.00	100.00	0.00	0.00
*Ficus sycomorus* L	33.09	54.68	12.23	38.85	21.58	39.57
*Flacourtia indica* (Burm. f.) Merrill	66.67	33.33	0.00	0.00	100.00	0.00
*Flueggea virosa* (Roxb. ex Willd.) Voigt	33.33	50.00	16.67	66.67	16.67	16.67
*Gardenia erubescens* Stapf & Hutch	39.25	51.40	9.35	43.93	19.63	36.45
*Gardenia ternifolia* Schumach. & Thonn	100.00	0.00	0.00	100.00	0.00	0.00
*Gmelina arborea* Roxb. [cult.]	62.50	37.50	0.00	62.50	25.00	12.50
*Grewia bicolor* Juss	50.00	50.00	0.00	50.00	0.00	50.00
*Grewia cissoides* Hutch. & Dalziel	0.00	100.00	0.00	0.00	0.00	100.00
*Grewia lasiodiscus* K.Schum	20.00	40.00	40.00	80.00	0.00	20.00
*Grewia* spp	0.00	100.00	0.00	100.00	0.00	0.00
*Guiera senegalensis* J.F.Gmel	19.05	76.19	4.76	38.10	0.00	61.90
*Gymnosporia senegalensis* (Lam.) Loes	69.23	15.38	15.38	53.85	7.69	38.46
*Haematostaphis barteri* Hook.f	25.00	75.00	0.00	25.00	0.00	75.00
*Hibiscus cannabinus* L. [cult.]	14.29	28.57	57.14	57.14	0.00	42.86
*Hymenocardia acida* Tul	22.22	77.78	0.00	66.67	11.11	22.22
*Hyparrhenia* spp	50.00	50.00	0.00	50.00	0.00	50.00
*Hyptis spicigera* Lam	22.22	51.39	26.39	6.94	33.33	59.72
*Indigofera bracteolata* DC	100.00	0.00	0.00	50.00	0.00	50.00
*Ipomoea carnea* Jacq	0.00	100.00	0.00	50.00	0.00	50.00
*Isoberlinia doka* Craib & Stapf	37.25	54.90	7.84	41.18	7.84	50.98
*Jatropha curcas* L. [cult.]	66.67	18.52	14.81	7.41	11.11	81.48
*Khaya senegalensis* (Desv.) A. Juss	29.24	49.12	21.64	45.61	13.45	40.94
*Kigelia africana* (Lam.) Benth	50.00	50.00	0.00	80.00	10.00	10.00
*Landolphia heudelotii* A. DC	71.43	28.57	0.00	28.57	42.86	28.57
*Lannea acida* A. Rich	54.29	31.43	14.29	54.29	17.14	28.57
*Lannea microcarpa* Engl. & K. Krause	25.53	60.64	13.83	34.57	23.94	40.96
*Lannea velutina* A.Rich	0.00	100.00	0.00	0.00	100.00	0.00
*Leptadenia hastata* (Pers.) Decne	50.00	37.50	12.50	62.50	12.50	25.00
*Loeseneriella africana* (Willd.) N.Hallé	0.00	100.00	0.00	0.00	0.00	100.00
*Lophira lanceolata* Tiegh. ex Keay	68.89	28.89	2.22	42.22	15.56	42.22
*Loudetia simplex* (Nees) C.E.Hubb	0.00	0.00	100.00	0.00	0.00	100.00
*Loudetia togoensis* (Pilg.) C.E.Hubb	0.00	0.00	100.00	0.00	0.00	100.00
*Maerua angolensis* DC	83.33	16.67	0.00	83.33	16.67	0.00
*Maerua crassifolia* Forssk	100.00	0.00	0.00	100.00	0.00	0.00
*Mangifera indica* L. [cult.]	68.03	24.59	7.38	45.08	23.77	31.15
*Mimosa pigra* L	16.67	50.00	33.33	16.67	0.00	83.33
*Mitragyna inermis* (Willd.) Kuntze	63.33	33.33	3.33	56.67	23.33	20.00
*Moringa oleifera* L	70.00	24.29	5.71	50.00	20.00	30.00
*Nymphaea lotus* L	57.14	14.29	28.57	57.14	0.00	42.86
*Ocimum americanum* L	8.70	34.78	56.52	10.87	6.52	82.61
*Oncoba spinosa* Forssk	50.00	33.33	16.67	50.00	38.89	11.11
*Opilia amentacea* Roxb	46.67	33.33	20.00	53.33	13.33	33.33
*Oxytenanthera abyssinica* (A.Rich.) Munro	100.00	0.00	0.00	100.00	0.00	0.00
*Ozoroa obovata* (Oliv.) R.Fern. & A.Fern	0.00	100.00	0.00	100.00	0.00	0.00
*Parinari curatellifolia* Planch. ex Benth	50.00	50.00	0.00	100.00	0.00	0.00
*Parkia biglobosa* (Jacq.) R. Br. ex G. Don f	29.33	50.96	19.71	41.35	16.83	41.83
*Paullinia pinnata* L	28.57	42.86	28.57	28.57	14.29	57.14
*Pennisetum pedicellatum* Trin	50.00	0.00	50.00	25.00	0.00	75.00
*Pennisetum* spp	12.00	28.00	60.00	24.00	4.00	72.00
*Pericopsis laxiflora* (Benth. ex Bak.) van Meeuwen	20.00	73.33	6.67	33.33	6.67	60.00
*Philenoptera laxiflora* (Guill. & Perr.) Roberty	38.89	61.11	0.00	22.22	0.00	77.78
*Piliostigma reticulatum* (DC.) Hochst	56.52	17.39	26.09	60.87	17.39	26.09
*Piliostigma thonningii* (Schum.) Milne-Redhead	44.12	35.29	20.59	42.65	20.59	36.76
*Prosopis africana* (Guill. & Perr.) Taub	50.00	40.91	9.09	31.82	36.36	31.82
*Pseudocedrela kotschyi* (Schweinf.) Harms	21.74	56.52	21.74	30.43	17.39	52.17
*Psidium guajava* L. [cult.]	100.00	0.00	0.00	61.90	14.29	23.81
*Pterocarpus erinaceus* Poir	32.32	56.10	11.59	43.90	21.95	34.15
*Pterocarpus santalinoides* DC	100.00	0.00	0.00	100.00	0.00	0.00
*Raphionacme splendens* Schltr	50.00	50.00	0.00	0.00	0.00	100.00
*Rottboellia cochinchinensis* (Lour.) Clayton	28.33	45.00	26.67	5.00	51.67	43.33
*Rytigynia senegalensis* Blume	0.00	0.00	100.00	0.00	0.00	100.00
*Saba senegalensis* (A. DC.) Pichon	30.22	58.27	11.51	33.09	29.50	36.69
*Sarcocephalus latifolius* (Sm.) E.A.Bruce	39.47	39.47	21.05	57.89	5.26	36.84
*Sclerocarya birrea* (A. Rich.) Hochst	76.19	23.81	0.00	57.14	14.29	28.57
*Securidaca longipedunculata* Fresen	65.85	26.83	7.32	70.73	7.32	21.95
*Senegalia ataxacantha* (DC.) Kyal. & Boatwr	16.67	50.00	33.33	16.67	33.33	50.00
*Senegalia gourmaensis* (A.Chev.) Kyal. & Boatwr	4.76	66.67	28.57	33.33	4.76	61.90
*Senegalia macrostachya* (Reichenb. ex DC.) Kyal. & Boatwr	30.00	30.00	40.00	60.00	0.00	40.00
*Senegalia polyacantha* (Willd.) Seigler & Ebinger	57.14	42.86	0.00	14.29	28.57	57.14
*Senegalia senegal (*L.) Britton	0.00	100.00	0.00	0.00	0.00	100.00
*Sporobolus pyramidalis* P.Beauv	33.33	50.00	16.67	50.00	0.00	50.00
*Sterculia setigera* Delile	64.86	29.73	5.41	48.65	18.92	32.43
*Stereospermum kunthianum* Cham	28.57	42.86	28.57	42.86	0.00	57.14
*Striga hermonthica* (Delile) Benth	41.18	41.18	17.65	0.00	72.55	27.45
*Strophanthus hispidus* DC	57.14	42.86	0.00	57.14	0.00	42.86
*Strychnos innocua* Delile	75.00	25.00	0.00	75.00	25.00	0.00
*Strychnos spinosa* Lam	21.54	64.62	13.85	41.54	10.77	47.69
*Tamarindus indica* L	31.40	54.55	14.05	47.93	14.05	38.02
*Tapinanthus* spp	68.42	31.58	0.00	31.58	5.26	63.16
*Tectona grandis* L.f. [cult.]	72.00	28.00	0.00	26.00	28.00	46.00
*Tephrosia linearis* (Willd.) Pers	33.33	0.00	66.67	0.00	33.33	66.67
*Terminalia avicennioides* Guill. & Perr	29.03	61.29	9.68	70.97	9.68	19.35
*Terminalia engleri* Gere & Boatwr.	46.67	33.33	20.00	46.67	20.00	33.33
*Terminalia macroptera* Guill. & Perr	58.97	41.03	0.00	41.03	35.90	23.08
*Terminalia schimperi* Hochst. ex Hutch. & Dalziel	29.63	55.56	14.81	38.27	22.22	39.51
*Trichilia emetica* Vahl	66.67	25.00	8.33	41.67	0.00	58.33
*Triumfetta lepidota* K.Schum	30.77	61.54	7.69	46.15	0.00	53.85
*Vachellia nilotica* (L.) P.J.H.Hurter & Mabb	84.21	13.16	2.63	52.63	21.05	26.32
*Vachellia seyal* (Del.) P.J.H.Hurter	0.00	100.00	0.00	0.00	0.00	100.00
*Vachellia sieberiana* (DC.) Kyal. & Boatwr	50.00	28.57	21.43	35.71	14.29	50.00
*Vernonia colorata* (Willd.) Drake	62.50	31.25	6.25	50.00	6.25	43.75
*Vitellaria paradoxa* C.F. Gaertn	18.80	46.15	35.04	29.49	20.51	50.00
*Vitex chrysocarpa* Planch. ex Benth	87.50	12.50	0.00	50.00	25.00	25.00
*Vitex doniana* Sweet	31.07	57.28	11.65	42.72	10.68	46.60
*Ximenia americana* L	43.96	52.75	3.30	46.15	14.29	39.56
*Zanthoxylum zanthoxyloides* (Lam.) Zepernick & Timler	45.45	54.55	0.00	45.45	9.09	45.45
*Ziziphus mauritiana* Lam	41.67	50.00	8.33	75.00	8.33	16.67
*Ziziphus mucronata* Willd	0.00	100.00	0.00	100.00	0.00	0.00

The population dynamics of the species are generally conceived as static because the proportion of opinions in favor of a decrease in species (40.08%) was approximately equal to that of the opinions in favor of their increase (40.86%). However, only 19.06% stated a constancy (Table 5). Among the most used species, *V. paradoxa* is the species with the highest positive dynamics (50% increase and 20.51% constancy) while *A. digitata* is the one with the highest negative dynamics (52.3% decrease).

### Perception of conservation of Sudanian savanna ecosystems by local populations

Regarding the variables leading to vegetation degradation, local populations agree that fire, cutting of fresh wood, and clearing for extension of cultivated areas were most important. In terms of importance, the majority of the sample ranked fire as the first cause of ecosystem degradation. However, for the Dagara of TWRB, the Kassena, and the Mossi of GRN, demographic increase is the most important cause (Table 6).

**Table 6:** Average score and rank of causes of ecosystem degradation among different groups in the sample

Informants	Fire	Wood cutting	Clearing	Demographic increase	Pasture	Climate change	Forest management
Global	**5.04**	*4.88*	***4.73***	4.48	3.48	3.21	2.13
Age classes							
Young	**5.20**	***4.83***	*4.89*	4.33	3.72	3.17	1.84
Adult	**5.09**	*4.92*	***4.43***	4.52	3.28	3.48	2.18
Elders	4.66	*4.91*	**5.04**	***4.68***	3.43	2.75	2.53
Sociocultural groups							
Dagara_B	*4.92*	4.30	***4.68***	**5.02**	3.60	2.80	2.32
Dagara_D	**5.70**	*5.48*	***4.80***	2.76	3.28	3.06	2.92
Pougouli	**5.05**	***4.75***	*4.80*	4.70	2.90	2.90	2.90
Mossi_B	**5.35**	*5.20*	***4.85***	4.60	2.50	3.40	2.10
Mossi_N	*4.74*	***4.64***	4.60	**4.94**	3.72	3.72	1.64
Kassena	4.66	*5.04*	***4.78***	**5.08**	3.92	3.30	1.36
Gender							
Man	*4.94*	**5.01**	***4.85***	4.45	3.40	3.43	2.19
Women	**5.13**	*4.76*	***4.62***	4.52	3.55	2.98	2.08

Emphasis: Bold—1st rank, italic—2nd rank, bold italics—3rd rank; Sociocultural groups: Dagara_B: Dagara of TWRB; Dagara_D: Dagara of CAD; Mossi_N: Mossi of GRN; Mossi_B: Mossi of TWRB

Local populations ranked, by order of preference, five solutions for ecosystem conservation and four motivations for participating in sustainable management of ecosystems. For the suggested solutions, they ranked first "raising the awareness of local populations of the danger from degradation of natural resources," followed by "prohibition of fires by forest authorities" and “subsidy by the government” (Table 7). These solutions relate to a participation of local populations in the management of plant resources. The majority of the sample place awareness raising as the primary solution. However, the Pougouli, the Dagara of CAD, and the women place fire prohibition as the first solution, while the Dagara of TWRB place government subsidies as the first solution.

**Table 7:** Average score and rank of suggested solutions for ecosystem conservation among different groups in the sample

Informants	Awareness of degradation	Fire prohibition	Subsidy from the government	Inspection and reforestation	Inclusive management
Global	**2.27**	*1.75*	***1.42***	1.28	0.95
Age classes					
Young	**2.25**	*1.98*	***1.40***	1.08	0.80
Adult	**2.43**	***1.62***	*1.65*	1.16	0.94
Elders	**2.00**	***1.64***	1.04	*1.81*	1.23
Sociocultural groups					
Dagara_B	*2.40*	0.92	2.78	***1.46***	1.20
Dagara_D	*2.84*	**3.52**	0.80	***1.30***	0.26
Pougouli	*2.25*	**2.45**	0.30	***1.55***	1.40
Mossi_B	**2.05**	***1.00***	*1.60*	0.75	0.50
Mossi_N	**1.92**	*1.40*	***1.14***	1.04	0.76
Kassena	**2.02**	1.20	1.34	***1.40***	*1.58*
Gender					
Men	**2.44**	1.20	*1.64*	***1.33***	1.28
Women	*2.10*	**2.31**	1.20	***1.22***	0.62

Emphasis: Bold—1st rank, italic—2nd rank, bold italics—3rd rank; Sociocultural groups: Dagara_B: Dagara of TWRB; Dagara_D: Dagara of CAD; Mossi_N: Mossi of GRN; Mossi_B: Mossi of TWRB

The motivation of local populations for sustainable management of plant resources is preferentially linked to their overall well-being due to the continuity of the provision of ES which will be ensured by long-term presence of the species, followed by the consideration of basic personal needs such as health, basic education, drinking water, and electricity provision by local authorities through the development of the village. Sustainability of vegetation is placed as the first motivation for sustainable management of plant resources by the majority of the sample. However, the elders, the Pougouli, the Mossi of GRN, and the men place village development as primary motivation (Table 8).

**Table 8:** Average score and rank of motivations for ecosystem conservation among different groups in the sample

Informants	Vegetation sustainability	Village development	Diversification of income	Obtaining a Job
Global	**2.96**	*2.93*	***2.49***	1.65
Age classes				
Young	**3.03**	*3.02*	***2.44***	1.59
Adult	**2.86**	*2.75*	***2.61***	1.76
Elders	*3.02*	**3.11**	***2.34***	1.53
Sociocultural groups				
Dagara_B	**3.00**	*2.80*	***2.14***	2.06
Dagara_D	**2.96**	***2.56***	*2.68*	1.80
Pougouli	*2.65*	**3.10**	***2.50***	1.75
Mossi_B	**3.10**	**2.75**	*2.80*	1.20
Mossi_N	*2.90*	**3.36**	***2.52***	1.38
Kassena	**3.04**	*3.00*	***2.48***	1.48
Gender				
Men	*2.88*	**3.00**	***2.35***	1.89
Women	**3.03**	*2.86*	***2.63***	1.40

Emphasis: Bold—1st rank, italic—2nd rank, bold italics—3rd rank; Sociocultural groups; Dagara_B: Dagara of TWRB; Dagara_D: Dagara of CAD; Mossi_N: Mossi of GRN; Mossi_B: Mossi of TWRB

## Discussion

### Diversity of utilized plant species and ecosystem services

Altogether, 163 plant species were cited by local populations as those providing them with different ecosystem services from Sudanian savannas. When considering the total number of species (1410) found by Zizka et al. [10] in the South Sudanian phytogeographic sector of Burkina Faso, only 11.6% of the potential flora of the study area are used by local populations. Each ES involves a great diversity of plant species: at least 60 species are used for 10 ES. This diversity of used resources makes it possible to overcome the problem of insufficient plant resources for a given service and could be exploited for the substitution of the most threatened species by those having a sufficiently high abundance in vegetation [23]. However, some specific ES involve specific species with specific property and characteristic. For example, the construction service is provided by stable and resistant species such as *Khaya senegalensis* (Desv.) A. Juss., *Anogeissus leiocarpa*, *Burkea africana* Hook. and *Pterocarpus erinaceus* Poir. [5]. In the study area, the most cited services were food supply followed by medicinal services. The importance of both services has been reported from investigations in the West [40], South [41] and North [35] of Burkina Faso and Côte d’Ivoire [42].

The relatively high percentage of use of fruits and leaves could be explained by their importance in various services such as food, fodder supply and medicinal use. The high frequency of fruit citation (70%) for food supply shows the importance of fruit in the diet of local populations. Wild fruits contribute to a varied diet in terms of vitamin (A, B, C, D, and E) and micronutrient intake [41, 43]. For example, the content of vitamin C in fruit of *Adansonia digitata* and *Detarium microcarpa* is as high as in orange fruit; *Moringa oleifera* contains twice as much protein as yogurt. In addition, plant species used for food supply were also used for medicinal service. Sourabié et al*.* [44] reported anti-diarrheal effects of the fruit’s pulp of *Adansonia digitata* and lowering of hypertension by *Parkia biglobosa* seeds.

The highest relative frequency (31%) of citations of the whole plant shows that local people are aware of the importance of vegetation and trees for their well-being, as the services they associate with the whole plant are regulatory, cultural, and supporting. These services are not destructive for plants and ecosystems but are rather conservative. This demonstrates local populations being committed to the conservation of their environment which constitutes their living space.

The 10 species with the highest use values were all woody species, and the shea tree (*Vitellaria paradoxa*) is a very popular species with the highest use value (3.775). In addition to its use in almost all services (14 of 15), it has a real use (UV) far more important than that of other species and a high socioeconomic value. This oleaginous species represents the fourth exportation product of Burkina Faso after gold, cotton, and livestock. Its high importance for populations was confirmed by other ethnobotanical studies [6, 24, 40, 41, 45, 46]. Almonds and fruits of *Vitellaria paradoxa*, fruit pulp and seeds of *Parkia biglobosa*, and fruit pulp and leaves of *Adansonia digitata* are highly appreciated as non-timber forest products which provide income through their trade [47]. Shea butter (from *Vitellaria paradoxa)* and the African mustard, also called soumbala (from *Parkia biglobosa*), are transformed products with strong chains of added values [48, 49]. *Diospyros mespiliformis* and *Lannea microcarpa* have highly appreciated edible fruits and medicinal uses. *Faidherbia albida* is a fodder woody species most appreciated by livestock breeders because it bears leaves and pods during the dry season when most of the woody species have shed leaves and fruits [35]. *Faidherbia albida* also has a high value of fertilizing cultivated or fallow soils [33] and is seen as a mystic plant by the Mossi [50]. *Khaya senegalensis, Ficus sycomorus, Afzelia africana* and *Pterocarpus erinaceus* are also fodder species with medicinal and cultural value [20, 50, 51]. Anti-malarial effects of *Afzelia africana*, *Khaya senegalensis, Ficus sycomorus, Parkia biglobosa* and *Pterocarpus erinaceus* are reported from Ghana [52]. The medicinal uses of the species most cited from our study area are also mentioned from other regions of Burkina Faso [35, 44, 53].

With regard to the high use value and the high demand for the products of most cited species, they constitute key species for local populations according to the definition by Clark and Sunderland [54]. However, once the value of a NTFP and the intensity of its use are extremely high, the resource is very likely to be overexploited, causing it to become locally extinct [54]. Gaisberger et al*.* [55] showed that overexploitation of species has emerged as the most important short-term threat. Overexploitation is the single most important threat for *Parkia biglobosa* (41.2%) and *Vitellaria paradoxa* (41.1%), and is only slightly exceeded by climate change in the case of *Adansonia digitata* (38.0%). The ethno-botanical use values correlated with the number of uses identified for each species and revealed the species preferred by local populations. However, the results must be taken with caution as the applied method does not distinguish between past, present and potential uses (some species may disappear because of anthropogenic pressure) [45, 56].

### Variables influencing the knowledge of ecosystem services

The three levels of land use intensity of this study design account for the economic benefit that local communities derive from them. The populations of the CAD and the TWRB live mainly from agriculture. The populations living near the TWRB engage in illegal activities such as farming, pasture and wood cutting in the protected area [14] as they do not benefit from economic benefits of the protected area in the same way as those of GRN. In fact, at least 18% of TWRB had been cleared [57] by local populations to install their fields. Forest administration has great difficulties to prevent neighboring villagers from using the resources in protected zones that they highly depend on [7, 13] as long as no incentives are offered for compensation. In contrast, inhabitants living near GRN are employed with the forest officers to ensure functioning of this tourist attraction and vary their income by participating in forest management. Likewise, hunting in the village hunting zone around GRN and fishing in water points of the ranch provide populations with additional income. Therefore, providing biodiversity conservation actors with diversified sources of income is economically important to local communities [15, 58]. The management of natural resources involving local communities contributes to better security of biodiversity through sustainable participatory management [59].

As for the sociocultural groups, the Pougouli and Dagara had more knowledge in ES referring to food supply, religion, wind protection, shading, medicinal use, soil fertilization, erosion prevention and energy supply. Their knowledge in religion services expresses the animist cult influence of Pougouli. According to the national statistics of population, the populations of the Southwest region have 64.9% of animists [60]. The high rate of religion ES is a means to preserve the surrounding vegetation and ecosystem. Religions are excellent channels for transmitting local knowledge through initiations (i.e., traditional and spiritual instructions) which are part of the education of the youngest in the preservation of the environmental values. To the animist communities such as Pougouli and Dagara, forests are the habitats of venerate spirits [50, 51]. The good knowledge about species providing medicinal services may be explained by the preservation of their ancestral knowledge transmitted from generation to generation [3, 16, 61]. The use of species for food supply, wind protection, shading and energy supply ES reflects the poverty of these populations who heavily depend on income from agriculture and small livestock. The Dagara and Pougouli are introvert sociocultural groups little open to exterior influence and quite attached to their local environment. Pougouli and Dagara knowledge is also orientated to performing farming. They are essentially farmers and do not hesitate to transgress protected areas to install their fields [14]. The population of GRN pays little attention to medicinal services due to the fact that they live nearby a health center, leading to a loss of local knowledge about medicinal plants. Local populations are subjected to increasing social (demographic and economic) and environmental pressures which have mostly led to a loss of knowledge [24, 62]. TWRB Mossi had no specific knowledge about ES provided by Sudanian savanna species. The lack of specific knowledge about Sudanian vegetation of the Mossi migrants of TWRB could be related to the fact of having immigrated from the Sudano-Sahelian zone where the species composition is different. The ethnobotanical knowledge varies, effectively, across sociocultural groups because of cultural differences and social habits [4, 63]. In addition, the migrant communities of TWRB are located outside the natives’ villages which constitutes an obstacle for passing on and sharing inter-community knowledge.

On the contrary, the Kassena and GRN Mossi sociocultural groups possessed similar knowledge and had more knowledge in craft, energy and touristic services than the Mossi of TWRB, the Native of TWRB and CAD. This demonstrates that the Mossi migrants of GRN are well integrated and adapted to the native way of life. The Mossi migrants of GRN have replaced species which they originally used by species which serve the same purpose among the natives (the Kassena). Thus, the traditional use of species by migrant communities can be influenced more strongly by the environment than by cultural heritage [64].

### Vulnerability of the species

The multipurpose use of species, the utilization of slow regenerating plant organs (as wood, seeds, bark, roots and flowers), and the preference (the high use value) that populations have for some species act to increase their vulnerability. The 10 species with high use value were highly vulnerable, except for *Faidherbia albida* and *Vitellaria paradoxa* classified as moderately vulnerable*.* The state of vulnerability of *Adansonia digitata, Afzelia africana, Khaya senegalensis, Parkia biglobosa* and *Pterocarpus erinaceus* is confirmed by Thiombiano et al*.* [65] who classify them threatened species of the southern Sudanian zone. According to these authors, *Diospyros mespiliformis* would be more threatened in the northern Sudanian zone. In the sub-Sahelian zone, Ouedraogo et al*.* [35] confirm high vulnerability of *Adansonia digitata*, *Diospyros mespiliformis, Faidherbia albida*, *Khaya senegalensis*, *Lannea microcarpa, Parkia biglobosa*, *Pterocarpus erinaceus* and *Vitellaria paradoxa*. All the 10 most used species of this study are also considered endangered by local populations of northern Benin [66]. Nevertheless, for the vast majority of plant species in Burkina Faso insufficient data are available for a full IUCN assessment [11, 27]. Globally and according to the IUCN red list [67], the conservation status of *Pterocarpus erinaceus* has been decreasing and has become Endangered, indicating a high risk of extinction. *Afzelia africana, Khaya senegalensis* and *Vitellaria paradoxa* conservation status are classified Vulnerable, indicating risk of extinction. The status of *Parkia biglobosa*, *Diospyros mespiliformis*, *Adansonia digitata*, *Lannea microcarpa, Faidherbia albida* and *Ficus sycomorus* is Least Concern. Although these species have different levels of vulnerability in other regions of Burkina Faso [11, 35] due to utilization by local populations, they are of great interest to the populations of our study sites. It would then be appropriate to think about their sustainable management through rational use motivated by the perceptions of local populations.

### Local perceptions about availability and population dynamics of the most used species

Perceptions of local populations on the availability and population dynamics of used species follow the same trend in the study area. Local populations stated that *Vitellaria paradoxa* was the most abundant species and showed an increase, whereas *Adansonia digitata* was very rare and showed a decrease. Traoré et al. [20] found that, in the Southwest of the country, perception of the state of resources by the local Senufo is consistent with the findings in the field. Indeed, 64% of the species cited by the Senufo as being rare are part of the rare species revealed by the calculation of the rarity index at the end of floristic inventories. Thus, the least variation in the availability of important species is perceived by local populations. The socioeconomic importance, availability and population dynamics of species define the management of traditional agroforestry systems. According to Assogbadjo et al. [21], species perceived by local communities as threatened are integrated into traditional agroforestry systems. However, the populations admit general degradation of the ecosystems.

### Local perception of conservation of Sudanian savanna ecosystems

According to local populations, fire, deforestation and clearing constitute the three main causes of Sudanian vegetation degradation. Local populations use fire as a tool for hunting, clearing of village surroundings and field preparation [63]. Deforestation by wood cutting results from the demand for energy, craft and construction services. The clearing of natural vegetation is practiced for installing new and extending existing agricultural land. Local populations are conscious of the causes of the degradation of plant resources, being essentially them provoking ecological pressure originating from increasing needs of growing populations [7, 13, 14]. While the development of agriculture has made it possible to free oneself from dependence on wild food, the expansion of agricultural land strongly contributes to ecosystem and biodiversity degradation [68]. Land use changes effectively have a negative impact on biodiversity due to habitat loss or fragmentation [20]. In the area of high land use intensity (CAD), species diversity is low and only species which provide ES are conserved.

Raising the awareness of local populations in the face of degrading natural resources is the first solution unanimously suggested by the communities. Given that climate change is not well perceived at the local scale, awareness raising may contribute to reducing anthropogenic pressure (fire, wood logging). Local population’s awakening of awareness as to the vulnerability of plant resources used every day and their implication for natural resources management in collaboration with forest authorities are steps that would enhance sustainable conservation of plants. The success of biodiversity conservation efforts often depends on local populations, especially when these communities are the key players in ecosystem management [69]. In fact, high biodiversity existing in native territories around the world is the result of traditional knowledge and management practices [16]. Fair collaboration of local populations with the forest authority for conservation of plant resources could be achieved, for example, by meeting certain basic needs such as the creation of water reservoirs and the construction of health centers and schools. Sustainability of vegetation allows for diversification of incomes, especially for women who collect and sell NTFP to complete their incomes [24]. Local processing of NTFP by women before selling contributes to the value chain of products and generating incomes. For example, the almond of *Vitellaria paradoxa* is transformed into shea butter and the seed of *Parkia biglobosa* is transformed into soumbala (African mustard) [48, 49].

## Conclusion

This study has highlighted the importance of ES of Sudanian savannas and the importance of long-term preservation of numerous plant species. It shows that local populations do know their environment well and that they are aware of the causes of degradation of plant resources. Therefore, local people should be involved in programs and projects addressing sustainable management and conservation of Sudanian savanna ecosystems. The behavioral change can be achieved by awareness raising and education. Education of local populations needs to involve best management strategies and promotion of domestication and regeneration of local multipurpose species. Also species with low utilization value should be protected for conserving future biological resources because species being less important today could be sought for in the future to replace others having become rare in the meantime.

However, in face of local population vulnerability and their strong dependence on natural resources, it is often difficult to consolidate conservation and rational and sustainable use of biodiversity. In order to achieve effective awareness raising among local populations for sustainable management of plant resources, it would be necessary to offer them solutions and motivations adapted to their perceptions and their consents. This approach will enable full collaboration of the sociocultural communities.

## Data Availability

The datasets used and/or analyzed in the current study are available from the corresponding author on reasonable request.

## Abbreviations

ES: Ecosystem services
TWRB: Total wildlife reserve of Bontioli (medium land use intensity)
CAD: Communal area of Dano (high land use intensity)
GRN: Game ranch of Nazinga (low land use intensity
